# Shared pedigree relationships and transmission of unreduced gametes in cultivated banana

**DOI:** 10.1093/aob/mcad065

**Published:** 2023-06-02

**Authors:** Guillaume Martin, Franc-Christophe Baurens, Karine Labadie, Catherine Hervouet, Frédéric Salmon, Franck Marius, Nilda Paulo-de-la-Reberdiere, Ines Van den Houwe, Jean-Marc Aury, Angélique D’Hont, Nabila Yahiaoui

**Affiliations:** CIRAD, UMR AGAP Institut, Montpellier F-34398, France; UMR AGAP Institut, Université Montpellier, CIRAD, INRAE, Institut Agro, Montpellier, France; CIRAD, UMR AGAP Institut, Montpellier F-34398, France; UMR AGAP Institut, Université Montpellier, CIRAD, INRAE, Institut Agro, Montpellier, France; Genoscope, Institut François Jacob, CEA, Université Paris-Saclay, Evry, France; CIRAD, UMR AGAP Institut, Montpellier F-34398, France; UMR AGAP Institut, Université Montpellier, CIRAD, INRAE, Institut Agro, Montpellier, France; UMR AGAP Institut, Université Montpellier, CIRAD, INRAE, Institut Agro, Montpellier, France; CIRAD, UMR AGAP Institut, F-97130 Capesterre-Belle-Eau, Guadeloupe, France; UMR AGAP Institut, Université Montpellier, CIRAD, INRAE, Institut Agro, Montpellier, France; CIRAD, UMR AGAP Institut, F-97130 Capesterre-Belle-Eau, Guadeloupe, FranceFrance; UMR AGAP Institut, Université Montpellier, CIRAD, INRAE, Institut Agro, Montpellier, France; CIRAD, UMR AGAP Institut, CRB-PT, F-97170 Roujol Petit-Bourg, Guadeloupe, FranceFrance; Bioversity International, Willem De Croylaan 42, B-3001, Leuven, Belgium; Génomique Métabolique, Genoscope, Institut François Jacob, CEA, CNRS, Université Evry, Université Paris-Saclay, Evry, France; CIRAD, UMR AGAP Institut, Montpellier F-34398, France; UMR AGAP Institut, Université Montpellier, CIRAD, INRAE, Institut Agro, Montpellier, France; CIRAD, UMR AGAP Institut, Montpellier F-34398, France; UMR AGAP Institut, Université Montpellier, CIRAD, INRAE, Institut Agro, Montpellier, France

**Keywords:** *Musa acuminata*, *Musa balbisiana*, *Musa schizocarpa*, banana cultivars, Mchare, parentage, unreduced gamete, polyploid, whole genome approach

## Abstract

**Background and Aims:**

Cultivated bananas resulted from inter(sub)specific hybridizations involving *Musa* species and subspecies (*M. acuminata* subspecies, *M. schizocarpa*, *M. balbisiana*) and the subsequent selection, centuries ago, of hybrids with parthenocarpic, seedless fruits. Cultivars have low fertility and are vegetatively propagated, forming groups of somaclones. Relatively few of them, mainly triploids, are grown on a large scale and characterization of their parental relationships may be useful for breeding strategies. Here we investigate parental relationships and gamete-type contributions among diploid and polyploid banana cultivars.

**Methods:**

We used SNP genotyping data from whole-genome sequencing of 178 banana individuals, including 111 cultivars, 55 wild bananas and 12 synthetic *F*_1_ hybrids. We analysed the proportion of SNP sites in accordance with direct parentage with a global statistic and along chromosomes for selected individuals.

**Key Results:**

We characterized parentage relationships for 7 diploid cultivars, 11 triploid cultivars and 1 tetraploid cultivar. Results showed that both diploid and triploid cultivars could have contributed gametes to other banana cultivars. Diploids may have contributed 1*x* or 2*x* gametes and triploids 1*x* to 3*x* gametes. The Mchare diploid cultivar group, nowadays only found in East Africa, was found as parent of two diploid and eight triploid cultivars. In five of its identified triploid offspring, corresponding to main export or locally popular dessert bananas, Mchare contributed a 2*x* gamete with full genome restitution without recombination. Analyses of remaining haplotypes in these Mchare offspring suggested ancestral pedigree relationships between different interspecific banana cultivars.

**Conclusions:**

The current cultivated banana resulted from different pathways of formation, with implication of recombined or un-recombined unreduced gametes produced by diploid or triploid cultivars. Identification of dessert banana’s parents and the types of gametes they contributed should support the design of breeding strategies.

## INTRODUCTION

Cultivated bananas (*Musa* sp.) resulted from a complex process of natural inter(sub)specific hybridization (Simmonds, 1962; Perrier *et al.*, 2011; Martin *et al.*, 2020*b*, 2023). They are vegetatively propagated and only a very limited number of these cultivars are grown on a large scale. For example, the commercial dessert Cavendish banana represents >50 % of the world production (Lescot, 2020). Banana culture faces major biotic stresses, including the fungal disease caused by a *Fusarium* sp. lineage named TR4, which currently threatens banana cultures (Viljoen *et al.*, 2020). There is thus a crucial need for breeding disease-resistant bananas while maintaining agro-morphological and fruit quality traits. However, breeding strategies are hampered by the sterility or very low fertility of cultivars, which limits crossing abilities and progeny number. In this context, knowledge of cultivar parentage would represent a very useful resource for breeding.

The main domestication traits of bananas relate to fruit edibility, with the selection by humans of plants with parthenocarpic seedless fruits, and therefore with low fertility. Three main species are involved in their formation process: *Musa acuminata* (A genome, 2*n* = 2*x* = 22), *M. balbisiana* (B genome, 2*n* = 2*x* = 22) and *M. schizocarpa* (S genome, 2*n* = *2x* = 22). So far, the A genome has been present in all analysed banana cultivars, except the small group of Fe’i cultivars that derived only from *Musa* species of the *Australimusa* series (T genome, 2*n* = 2*x* = 20) (Simmonds and Shepherd, 1955; Jarret *et al.*, 1992; Martin *et al.*, 2023).

Hybridizations involving *M. schizocarpa* and *M. acuminata* subspecies (ssp. *banksii* and possibly ssp. *zebrina*) in New Guinea are proposed as the starting point of banana domestication (Martin *et al.*, 2023). Along with diffusion of early cultivars throughout Southeast Asia, additional hybridizations occurred with other *Musa* species and subspecies, such as *M. acuminata* ssp. *zebrina*, *malaccensis*, *burmannica*, *halabanensis*, *M. balbisiana* and at least one unknown contributor that might be an uncharacterized *M. acuminata* subspecies (Martin *et al.*, 2020*b*, 2023; Sardos *et al.*, 2022). Large chromosomal rearrangements (i.e. translocation, inversion) present in some wild *Musa* were transmitted to many cultivars (Martin *et al.*, 2020*a*). This context of inter(sub)specific hybridization may have favoured production of 2*x* gametes from diploid hybrids, leading to the formation of triploid cultivars (Simmonds, 1962; Perrier *et al.*, 2011). The current diversity of cultivars includes diploids and triploids with different global genomic combinations (e.g. AA, AB, AAA, AAB, ABB, AAT) modulated by interspecific recombination and with an A genome introgressed by *M. schizocarpa* (Simmonds and Shepherd, 1955; Nemeckova *et al.*, 2018; Baurens *et al.*, 2019; Cenci *et al.*, 2021; Martin *et al.*, 2023). Within these genomic groups, cultivars are classified in distinct subgroups, each of which is thought to derive from one seed and centuries or millennia of vegetative propagation, and thus they represent subgroups of phenotypically different somaclonal mutants (Simmonds, 1954; Perrier *et al.*, 2011). The most-grown cultivar subgroups are triploid, including AAA dessert bananas such as Cavendish, AAA cooking East African Highland bananas and AAB plantain bananas.

The complex hybridization processes at the origin of cultivars would be difficult to rapidly reproduce in breeding programmes. The identification and use of direct parents of successful cultivars could thus be useful to facilitate banana breeding (Raboin *et al.*, 2005). Several parentages have been proposed based on analyses with low-density markers (RFLP and SSR) involving in particular the diploid Mchare cultivar subgroup (previously named Mlali) (Raboin *et al.*, 2005; Perrier *et al.*, 2009; Hippolyte *et al.*, 2012). Higher-density markers should allow confirmation of parentage relationships at whole-genome scale, and should provide additional information on cultivar pedigree and on gamete transmission processes.

The aim of this study was to use high-density single-nucleotide polymorphism (SNP) genotyping information from 178 banana individuals to identify potential parents of cultivars from our sample. Special emphasis was placed on the contribution of the Mchare cultivar subgroup and the parental relationships between Mchare-derived cultivars. This work also addresses questions on gamete transmission type leading to major cultivars and the potential role of triploids as parents to other triploids.

## MATERIALS AND METHODS

### Materials

A set of 178 banana individuals was first considered for this analysis (Supplementary Data Table S1). These individuals correspond to wild *Musa acuminata* (A genome, 2*n* = 2*x* = 22), *M. schizocarpa* (S genome, 2*n* = 2*x* = 22), *M. balbisiana* (B genome, 2*n* = 2*x* = 22) and *Australimusa* spp. accessions (T genome, 2*n* = 2*x* = 20), to cultivars derived from these species, and to 12 synthetic *F*_1_ hybrids generated at the CIRAD breeding platform by crosses between parents also represented in the dataset (Supplementary Data Table S1). SNP-based genotyping data (not phased) of these individuals were extracted from a larger vcf file obtained from high-coverage Illumina sequencing data used to perform chromosome ancestry painting, described in Martin *et al.* (2023). SNP positions are located along the DH-Pahang V4 reference genome (Belser *et al.*, 2021). All scripts used are available in the vcfhunter toolbox (https://github.com/SouthGreenPlatform/VcfHunter). Compared with the original file, a new genotype calling was performed (using TotalRecal.1.0.py), for accession DB_Pisang_Awak, which was tetraploid instead of triploid according to the Martin *et al.* (2023) analysis. Among the 178 individuals, 26 were similar to other accessions (i.e. somaclones or duplicates of other accessions). For these similar accessions (Supplementary Data Table S1), only one was reported in results tables. Representations of genome ancestry mosaic painting were published previously (Martin *et al.*, 2023; https://banana-genome-hub.southgreen.fr/node/50/1598445), except for newly generated results of deduced haplotypes (see below).

### Methods

Two types of SNP data analysis were performed (Supplementary Data Fig. S1). The first one consisted in the identification of triplets of individuals that match a direct parents–child trio relationship [i.e. the genotype from an individual (2*x*, 3*x* or 4*x*) defined as child is compatible with a combination of two individuals (2*x*, 3*x* or 4*x*) defined as parents when looking at their genotypes]. The second one consisted in the identification of pairs of individuals that match a direct parent–child duo relationship involving one individual from the diploid Mchare clonal subgroup as a parent [i.e. the genotype from an individual (2*x*, 3*x* or 4*x*) defined as child is compatible with Mchare as parent when looking at the genotype]. Both analyses were performed in two steps: (1) calculation of the global proportion of SNP sites in accordance with each tested trio or duo; and (2) validation of identified trios or duos through local analysis along chromosomes of the proportion of SNP sites in accordance with such trios or duos.

### Identification of potential parents–child trios through global SNP analysis

This analysis was performed using the ValPar.py tool, added to the vcfhunter toolbox. The tool worked as follows. (1) For each potential parent, all possible gametes were generated. These gametes were all combinations of *k* elements, with repetition and without order, sampled among *n* non-redundant alleles found at the studied position in the potential parent (*k* being the ploidy of the potential gamete). For example, for a position where a parent is ATT, the different possible diploid gametes are: AA, AT and TT. If the potential child was triploid, two values of *n* were tested for each parent, i.e. *n* = 1 and *n* = 2. (2) For each potential parent pair, all possible zygote allele combinations were generated by combining possible gametes from both parents (only gamete combinations giving the correct zygote ploidy were generated). (3) The allele combination found in the potential child was searched for among the possible zygote allele combinations from the parents. (4) The proportion of sites in which the potential child allele combination matched one of the possible zygote allele combinations from the parent pair was then calculated for each potential parent pair. Ploidy was considered; for example, if the potential child was triploid, two final values were reported: one with parent 1 being the 1*x* gamete donor and parent 2 being the 2*x* gamete donor, and a second one with parent 1 being the 2*x* gamete donor and parent 2 being the 1*x* gamete donor.

To save computation time, accessions without *Australimusa* ancestry were analysed using a filtered vcf including only polymorphic sites in the 167 accessions from *M. acuminata*, *M. schizocarpa* and/or *M. balbisiana* origin. The filtered vcf contained 6 867 490 polymorphic sites. Accessions with an *Australimusa* ancestry were analysed using a filtered vcf including only polymorphic sites in the 178 individuals (the 167 A, S and/or B accessions plus the 11 *Australimusa* or *Australimusa* hybrid accessions) (Supplementary Data Table S2). The filtered vcf contained 8 285 170 polymorphic sites.

The proportion of sites in accordance with tested parentage was calculated for each trio with a cultivated accession as a child (Supplementary Data Table S3). The following criteria were used to consider parental relationships as valid: (1) the proportion of sites in accordance with the tested parentage should be equal to or higher than 0.999, which is the minimal value observed in the 12 synthetic parents–child trios (Supplementary Data Table S4); and (2) among couples of parents that validated this threshold only those with the minimal cumulative ploidy were retained. The reason behind this is that we considered that diploids were more likely to be parents compared with triploids.

### Identification of potential Mchare contribution through global SNP analysis

The contribution of Mchare was specifically analysed by looking for a potential 1*x* and 2*x* Mchare gamete restitution on all individuals of the dataset.

According to Martin *et al*. (2023), Mchare ancestral contributors were only from *M. acuminata* ssp. and *M. schizocarpa*. Thus, only polymorphic sites between and within these genetic groups were kept for the analysis. For this, sites containing private alleles from other species (i.e. *M. balbisiana* or *Australimusa*; Supplementary Data Table S2) were identified and removed using IdentPrivateAllele.py, allele_ratio_group.py and vcfSelect.py as previously described (Martin *et al.*, 2023). If not removed, such sites would generate an important proportion of sites in accordance with the Mchare contribution only because they are homozygous in all individuals with *M. acuminata* and/or *M. schizocarpa* origin. The resulting vcf file contained 5 412 666 polymorphic SNP sites.

The analysis was then performed in several steps (Supplementary Data Fig. S1, purple steps): (1) selecting, for each duo (Mchare, potential child), only sites that are polymorphic within and/or between individuals of the duo using vcfFilter.1.0.py; (2) calculating the proportion of sites validating a potential 1*x* contribution of Mchare to the potential child using the ACRO.py tool added to the vcfhunter toolbox (Supplementary Data Table S5); (3) in case of a polyploid potential child, calculating the proportion of sites validating a potential 2*x* (recombined or un-recombined) contribution of Mchare to the potential child using the ACRO.py tool (Supplementary Data Table S5); (4) in case of a polyploid potential child, looking for the proportion of sites validating a complete Mchare genome (i.e. un-recombined 2*x* gamete, named hereafter 2*x*^c^) in the potential child using vcfRemove.1.0.py (Supplementary Data Table S5).

The summary statistics obtained from steps 2, 3 and 4 were used to identify potential duos with a Mchare contribution. For this, duos that had a proportion of SNP sites in accordance with a 1*x* or 2*x* contribution >99 % were selected (Supplementary Data Table S5). This value was selected based on an observed shift in the summary statistic values (Supplementary Data Table S5).

### Validation of parents–child trios through local SNP analysis along chromosomes

To validate selected parents–child trios, the distribution of the proportion of shared SNPs was inspected along reference chromosomes. This was performed in several steps (Supplementary Data Fig. S1, green steps), as follows. (1) Selecting, in the vcf file, the SNP sites that were polymorphic within and/or between individuals of the trio. (2) Calculating from the obtained vcf file the proportion of sites in accordance with the trio on windows of 201 SNPs size. Sites in accordance were calculated as described in the trio identification section above. (3) Filtering the vcf in order to keep, for each parent–child duo, polymorphic SNPs within and between individuals of the duo, and calculating from the resulting vcf file the proportion of sites in accordance with the duo on window sizes of 201 SNPs. This calculation considers the gamete ploidy of the tested parent. (4) In parallel, the number of alleles shared between the parent and the child was calculated to test for complete genome restitution. (5) In cases where the complete genome of the parent was found in the child, the alleles from this parent were removed from the child’s genotype in the vcf obtained in step (1). This allowed access to the gamete given by the other parent. (6) The remaining genotype(s) were compared with the genotype of the second potential parent to analyse the proportion of sites in accordance with such parentage. This analysis was performed using the APAR.py tool that was added to the vcfhunter toolbox.

For ease of representation, the proportions of sites in discordance with tested parentages were displayed along each chromosome and all results were visualized using Circos (Krzywinski *et al.*, 2009). Configuration and files required for Circos visualization were automatically generated by the APAR.py tool.

### Validation of parent–child duos through local SNP analysis along chromosomes

Validation of parent child duos (Supplementary Data Fig. S1, blue steps) was performed as follows: (1) for each duo, selection in the vcf of sites that were polymorphic within and between individuals of the duos using vcfFilter.1.0.py; (2) looking along chromosomes for the proportion of SNP sites validating a 1*x* contribution of the potential parent to the potential child using the ACRO.py tool; (3) in case of a polyploid potential child, looking along chromosomes for the proportion of SNP sites validating a potential 2*x* contribution of the potential parent to the potential child using the ACRO.py tool; (4) in case of a polyploid potential child, looking along chromosomes for the proportion of sites validating a complete parental genome in the potential child using the vcfIdent.1.0.py tool of the vcfhunter toolbox. These proportions were calculated along chromosomes on windows of size 201 SNPs.

The proportions of sites in discordance with tested parentages were represented along each chromosome and results were visualized using Circos (Krzywinski *et al.*, 2009) and the DrawCircos.py tool added to the vcfhunter toolbox.

### In silico *chromosome ancestry painting of remaining haplotype(s) in polyploid progeny where complete genome restitution of a parent was identified*

The genotype of the parent was removed from the polyploid child in the vcf using the vcfRemove.1.0.py tool. Remaining alleles were considered as a haplotype, representative of the gamete from the second parent. The *in silico* chromosome ancestry painting of the remaining haplotype(s) corresponding to the gamete from the second parent was performed using the ancestry specific alleles and the process described in (Martin *et al.*, 2023). The process was automated with the SPRH.py tool added to the vcfhunter toolbox (Supplementary Data Fig. S1).

Mosaics obtained for Mchare-derived AAB cultivars were compared with published banana mosaics (Martin *et al.*, 2023) by looking for similar ancestry mosaic patterns or compatible mosaics that could derive from recombination between parental haplotypes.

### Management of aneuploidy

The triploid Lady Finger (Nadan) accession had a supernumerary chromosome 8. To manage this aneuploidy, the genotype calling of Lady Finger along chromosome 8 was recalculated with a ploidy of 4 using the TotalRecal.1.0.py script of the vcfhunter toolbox. For this accession, the analyses described previously (with exception of the global one) were performed separately on triploid chromosomes and on chromosome 8 and results were pooled. For tetraploid Calypso, aneuploid regions were identified by analysing read coverage of SNP sites along chromosomes, using the vcf2cov.py program added to the vcfhunter toolbox.

## RESULTS

### Global parentage analysis identifies one parent or the two parents for several cultivars

Parentage relationships were searched among 178 banana individuals (Supplementary Data Table S1) using SNP genotyping data obtained by Martin *et al.* (2023) from whole-genome Illumina sequencing. The dataset included 55 wild accessions representing *M. acuminata* ssp. (A genome), *M. schizocarpa* (S genome), *M. balbisiana* (B genome) and *Australimusa* spp. (T genome), 111 cultivars derived from these species and 12 *F*_1_ diploid hybrids for which both parents were known. Banana cultivars belonging to a subgroup correspond to somaclonal variants that are currently indistinguishable at the genomic level. In order to facilitate the reading of the manuscript, when relevant, we will refer to the name of the subgroup (Supplementary Data Table S1) rather than to the name of the accession.

Ten parents–child trios were identified, as they showed a global proportion of SNP sites in accordance with the trio that was equal to or higher than the proportion (99.9 %) observed for the 12 *F*_1_ synthetic parents–child trios (Table 1, Supplementary Data Table S3). Three additional parents–child trios that involved *Australimusa* species were identified; however, two of them may correspond to clonal relationship between cultivars and the third to similarity between two wild accessions (Supplementary Data Table S3).

**Table 1.** Proposed banana parents–child trios and parent–child duos

							Gamete ploidy		Supported by local analysis
	Child	Child group	Parent 1 (P1)	P1 group	Parent 2 (P2)	P2 group	P1	P2	
Identified trios	IDN 077	AA	Sucrier	AA	Ibota	AAA	1*x*	1*x*	Yes
	Pisang Berlin	AA	Sucrier	AA	Tjau Lagada	AA	1*x*	1*x*	Yes
	Pisang Papan	AAA	Tjau Lagada	AA	Sucrier	AA	2x	1*x*	Yes
	Saba	ABB	Monthan	ABB	Pisang Klutuk Wulung	BB (wild)	2x	1*x*	Yes
	Colatina Ouro	AA	Mchare	AA	Sucrier	AA	1*x*	1*x*	Yes
	Mnalouki	AAB	Mchare	AA	Plantain	AAB	1*x*	2x	Yes
	Gros Michel	AAA	Mchare	AA	Khai Nai On	AA	2x^c^	1*x*	Yes
	*Galeo*	*AA*	*Palang*	*AAA*	*Sinwobogi*	*AA*	1*x*	1*x*	*Yes* ^***b***^
	*Galeo*	*AA*	*Palang*	*AAA*	*SF265*	*AA*	1*x*	1*x*	*Yes* ^***b***^
	*Galeo*	*AA*	*Khai Nai On*	*AA*	*Palang*	*AAA*	1*x*	1*x*	*Yes* ^***b***^
Identified additional duos	Cavendish	AAA	Mchare	AA	?	–	2x^c^	–	Yes
	Nadan	AAB	Mchare	AA	?	–	2x^c^	–	Yes
	Nendra Padaththi	AAB	Mchare	AA	?	–	2x^c^	–	Yes
	Pome	AAB	Mchare	AA	?	–	2x^c^	–	Yes
	Koja	AAA	Mchare	AA	?	–	1*x*	–	Yes
	Paka	AA	Mchare	AA	?	–	1*x*	–	Yes
	Pisang Ambon	AAA	Mchare	AA	?	–	1*x*	–	Yes
	*Calypso*	*AAAA*	*Mchare*	*AA*	*?*	*–*	*2x* ^*c*^	*–*	*Yes* ^***b***^
	Wh-O-Gu	AAA	Mchare	AA	?	–	1*x*	–	No
	Hom Thong Mokho	AAA	Mchare	AA	?	–	1*x*	–	No
Re-analysed^a^	Calypso	AAAA	Gros Michel	AAA	?	–	3x^c^	–	Yes
	Palang	AAA	Galeo	AA	?	–	2x^c^	–	Yes
	Khai Nai On	AA	Galeo	AA	?	–	1*x*	–	Yes
	Sinwobogi	AA	Galeo	AA	?	–	1*x*	–	Yes
	SF265	AA	Galeo	AA	?	–	1*x*	–	Yes

^a^Parentage re-analysed in section ‘Complete 2*x* and 3*x* gamete transmission among cultivated banana’ of Results.

^b^Indicates re-analysed, re-interpreted trios and duos (lines in italics).

^c^Indicates that a complete genome of the parent is found in the child.

The targeted search for parental contributions of the Mchare cultivar subgroup (Supplementary Data Table S5) yielded a total of 13 Mchare–child duos (Table 1). Three of these 13 duos (involving Gros Michel, Mnalouki, Colatina Ouro) were already identified among the parents–child trios. The duos involved two diploid, ten triploid and one tetraploid accessions. Among them, six accessions (five triploids and one tetraploid) had >99.6 % of sites in accordance with Mchare being the 2*x* gamete donor and >99.4 % of sites suggested that both haplotypes of Mchare are found in each of these six accessions (Supplementary Data Table S5).

### Analysis of SNPs along chromosomes validates most candidate parents from the parents–child trio and parent-child duo analysis

**Fig. 1.**
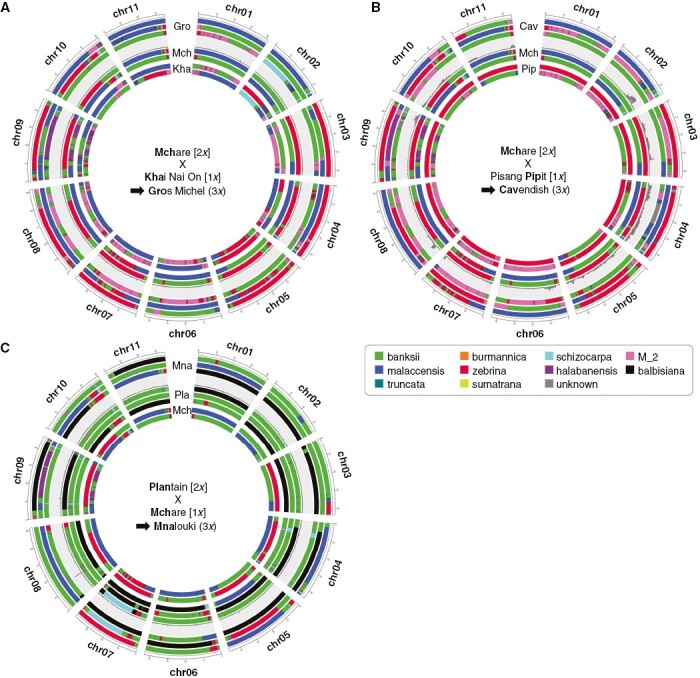
Validation along chromosomes of predicted parents–child trios. Accession chromosome ancestry mosaics obtained from (Martin *et al.*, 2023) and the local proportion of sites in discordance with tested trios are represented. Child pseudohaplotypes are represented on outer circles and are separated from tested parents’ pseudohaplotypes (on inner circles) by the local proportion of alleles in discordance with the tested parentage (value between 0 and 1). Ploidy of the parental tested gamete is indicated between square brackets and the ploidy of the child is indicated between round brackets. Colour codes ‘banksii’, ‘burmannica’, ‘zebrina’, ‘malaccensis’, ‘truncata’, ‘sumatrana’ and ‘halabanensis’ stand for an origin from *M. acuminata* ssp. *banksii/microcarpa/errans*, *burmannica*, *zebrina*, *malaccensis*, *truncata*, *sumatrana* and *halabanensis* respectively. ‘schizocarpa’ and ‘balbisiana’ stand for *M. schizocarpa* and *M. balbisiana* respectively. ‘M_2’ is an uncharacterized contributor to banana and ‘unknown’ corresponds to regions in which no origin could be attributed. Panels (A, B and C) correspond to three tested trios whose names are indicated in each figure centre.

The proposed parentages for cultivars (10 trios and 13 duos) were further investigated by local analysis of shared SNPs along their chromosomes. Two additional trios proposed in the literature (Perrier *et al.*, 2009; Hippolyte *et al.*, 2012) with Cavendish cultivars as a child, Mchare as 2*x* gamete donor and diploid cultivars Pisang Madu or Pisang Pipit as 1*x* gamete donor were also tested. The proportion of SNPs in discordance along the chromosomes was calculated and represented together with the *in silico* chromosomal ancestry painting (Fig. 1). Figure 1A represents the tested trio Gros Michel (child), Mchare (2*x* parent) and Khai Nai On (1*x* parent). The proportion of SNPs in discordance was close to zero along all chromosomes, validating this parents–child trio. In contrast, the two trios proposed in the literature with Cavendish as child showed large regions, on nearly all chromosomes, in which the proportion of SNPs in discordance was relatively high (Fig. 1B, Supplementary Data Fig. S2A), indicating that the direct trio parentages were not valid.

All ten trios suggested by the global analysis were validated by this approach (Fig. 1A, C, Supplementary Data Fig. S2B–I). For two of them, a peak of discordance was observed on a small region on chromosome 8 (Fig. 1C, Supplementary Data Fig. S2D), probably due to small differences (gene conversion, aneuploidy) resulting from divergence after vegetative propagation over centuries or problems of read mapping and variant calling. Among the ten validated trios, three distinct trios were proposed with Galeo as child (in italics in Table 1). This impossible situation is further investigated in the next section of results.

The seven remaining validated trios revealed different types of cross involving diploids and triploids as parents of diploid or triploid cultivars (Table 1, Fig. 1, Supplementary Data Fig. S2). Figure 1C illustrates a case where a plantain (AAB triploid genome) is proposed as a 2*x* gamete donor for another triploid cultivar. All identified parents are cultivars except in one case where a wild *M. balbisiana* accession [Pisang Klutuk Wulung (PKW)] is a parent of the triploid cultivar Saba (Table 1, Supplementary Data Fig. S2).

**Fig. 2.**
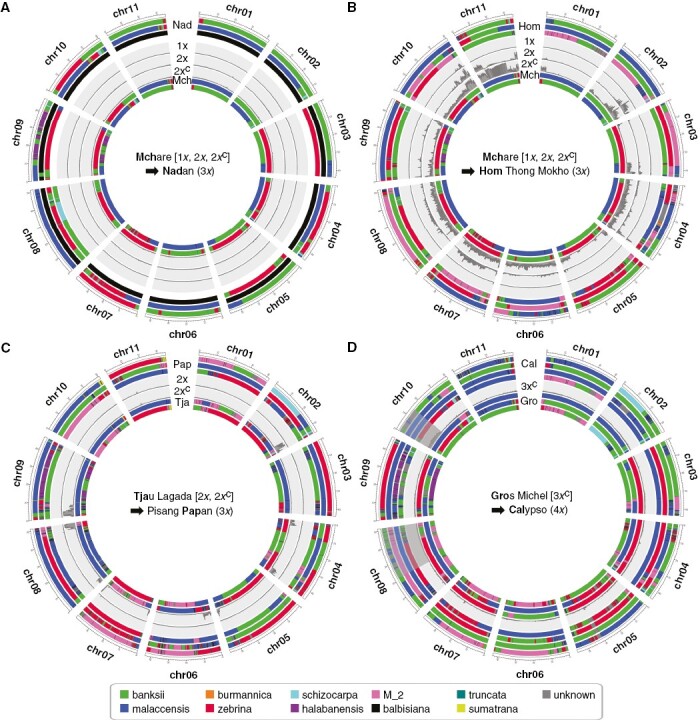
Validation along chromosomes of predicted parent–child duos and transmitted gamete types. Accession chromosome ancestry mosaics obtained from Martin *et al.* (2023) and the local proportion of sites in discordance with tested duos and gamete types are represented. Child pseudohaplotypes are represented on outer circles and are separated from tested parent pseudohaplotypes (on inner circles) by the local proportion of alleles in discordance with tested parentage (value between 0 and 1). Depending on the duos tested, different type of parentage could be tested: a haploid gamete restitution (1*x*), a diploid gamete restitution (2*x*), a diploid gamete with complete genome restitution (2*x*^c^) or a triploid gamete with complete genome restitution (3*x*^c^). Ploidy of the parental tested gamete is indicated between square brackets and the ploidy of the child is indicated between round brackets. Colour codes are identical to those in Fig. 1. Panels (A–D) correspond to four tested duos whose names are indicated in each figure centre.

Similar analyses were performed with the 13 predicted Mchare duos (including three already present in trios) and validated 11 of them (Table 1). A 2*x* contribution of the Mchare was validated for five triploid cultivars: Nadan (Fig. 2A), Gros Michel (Supplementary Data Fig. S3A), Cavendish (Supplementary Data Fig. S3B), Nendra Padaththi (Supplementary Data Fig. S3C) and Pome (Supplementary Data Fig. S3D). A 2*x* contribution of the Mchare was also found in tetraploid cultivar Calypso (Supplementary Data Fig. S3E). A 1*x* contribution of the Mchare was validated for two diploid cultivars [Paka (Supplementary Data Fig. S3F) and Colatina Ouro (Supplementary Data Fig. S3G)] and three triploid cultivars [Koja (Supplementary Data Fig. S3H), Pisang Ambon (Supplementary Data Fig. S3I) and Mnalouki (Supplementary Data Fig. S3J)]. In Pisang Ambon (Supplementary Data Fig. S3I), a small region at the beginning of chromosome 1 was discordant for a 1*x* Mchare contribution but corresponded to a region where Pisang Ambon is aneuploid with a ploidy of 2*x* instead of 3*x* (Martin *et al.*, 2023). The segment of Mchare origin was likely lost from this region during years of vegetative propagation of Pisang Ambon.

Finally, for two triploids, Hom Thong Mokho (Fig. 2B) and Wh-O-Gu (Supplementary Data Fig. S3K), regions of discordance, even with a 1*x* contribution of the Mchare were observed, suggesting that these accessions were not directly derived from Mchare accessions but are related to them.

### *Complete 2*x *and 3*x *gamete transmission among cultivated bananas*

The proportions of shared SNPs along chromosomes were analysed in the seven predicted cases of 2*x* gamete transmission by a diploid accession (Table 1) to specify the nature of the transmitted 2*x* gametes. One case involved the diploid Tjau Lagada, which transmitted a recombined 2*x* gamete to Pisang Papan (Fig. 2C). In six cases, a complete diploid Mchare genome restitution was detected (Fig. 2A, Supplementary Data Fig. S3A–E). These cases included the tetraploid accession Calypso, which is an improved cultivar derived from a cross between Highgate, a dwarf clone of the triploid Gros Michel cultivar and a diploid individual (Borges *et al.*, 2014). The Mchare contribution to Calypso identified in this duo is due to the presence of the Mchare genome in Gros Michel. The test of the complete genome restitution of Gros Michel along chromosomes of Calypso showed that only one region at one extremity of chromosome 10 was not in accordance with such complete genome restitution (Fig. 2D). Calypso is aneuploid for a few chromosomal regions, including a missing chromosomal region at one extremity of chromosome 10 (Fig. 2D, Supplementary Data Fig. S4). The results were thus in accordance with a complete 3*x* gamete restitution of Gros Michel to Calypso.

A particular situation was found for the Galeo diploid cultivar, which was proposed as child in three distinct trios involving the Palang triploid accession, as one parent, and any of the diploid accessions Khai Nai On, Sinwobogi or SF265 as the second parent (Table 1). These three diploid potential parents are not somaclones as they do not have the same ancestral mosaic genome (Martin *et al.*, 2023), which raised questions about the process generating these trios. Further examination of the trios involving Galeo revealed that the complete genome of Galeo was present in triploid Palang (Supplementary Data Fig. S5A). This led us to propose that Galeo may not be the child but rather the parent of these accessions and transmitted a 2*x* gamete to Palang and a 1*x* gamete to Khai Nai On, Sinwobogi and SF265 (Table 1). The first proposed trios are a consequence of the presence of the Galeo genome in Palang, which allows for recombinant haplotypes complementing those present in Khai Nai On, Sinwobogi and SF265 to generate Galeo (Supplementary Data Fig. S5B). Access to real haplotypes (phased data from genetic studies or long-read sequencing technologies) of these individuals should allow validation or rejection of this hypothesis.

### In silico *extraction and analysis of the remaining 1*x *gamete from Mchare-derived accessions*

**Fig. 3.**
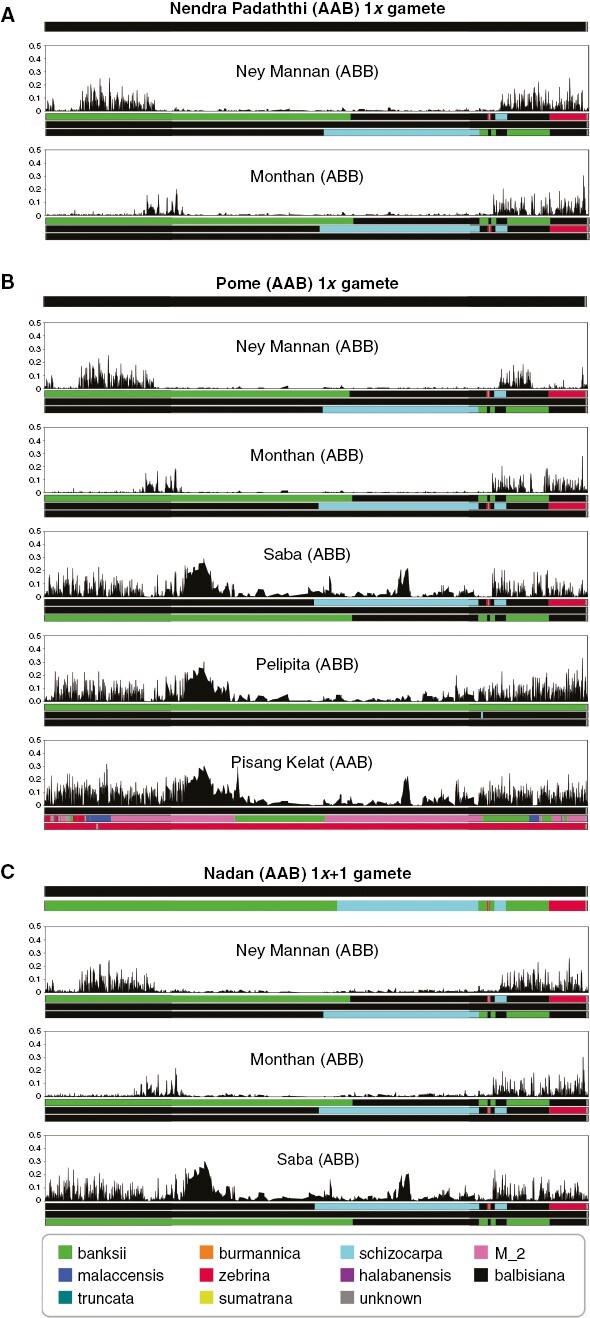
Patterns of local proportions of SNP sites in discordance with AAB/ABB tested parentage relationships on chromosome 8. Analysis was performed on deduced gametes from AAB Nendra Padaththi (A), Pome (B) and Nadan (C) after removal of the Mchare genotype that has been found complete in these individuals. These deduced gametes were compared with potential parents Ney Mannan and Monthan (A, B and C), Saba (B and C), Pisang Kelat and Pelipita (b), which showed compatible mosaics with deduced gametes. Black curves represent the local proportion of alleles in discordance with tested parentage (value between 0 and 0.5). Coloured horizontal bars at the top of each subfigure represent the deduced gamete ancestry mosaic for Nendra Padaththi (A), Pome (B) and Nadan (C). Horizontal bars under each curve represent the mosaic from the corresponding tested parent obtained from Martin *et al.* (2023). Colour codes are identical to those in Fig. 1.

**Fig. 4:**
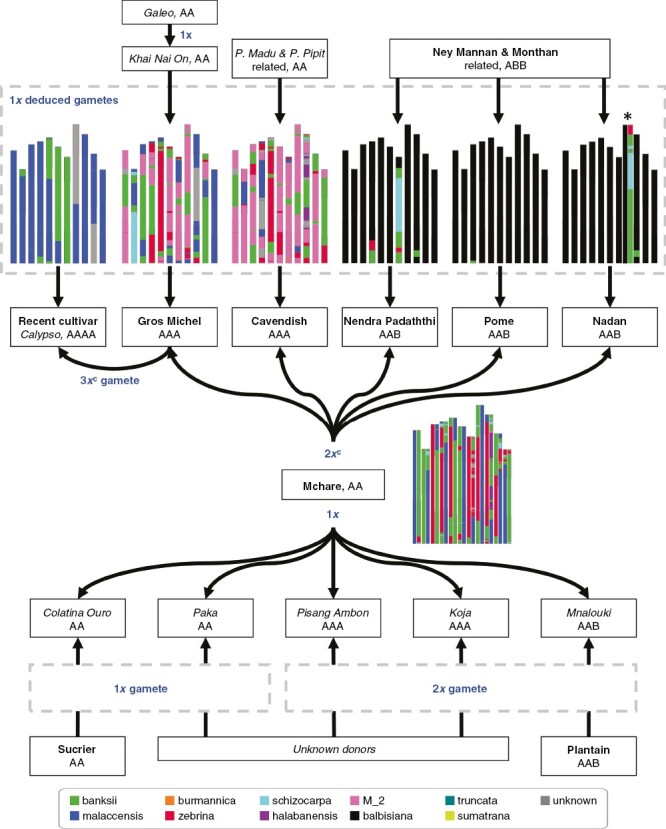
Contribution of Mchare to cultivated bananas and associated pedigree relationships. The contribution of identified parents is indicated by black arrows. The type of gamete contribution is indicated: 1*x*, haploid gamete restitution; 2*x*, diploid gamete restitution; 2*x*^c^, diploid gamete with complete genome restitution; 3*x*^c^, triploid gamete with complete genome restitution. Chromosome ancestry mosaics of Mchare (Martin *et al.*, 2023) and of the deduced gametes produced by the second parents of Mchare-derived cultivars are shown with bar plots. Colour codes are identical to those in Fig. 1. *Aneuploid chromosome 8 in Nadan.

For accessions derived from a complete gamete restitution (2*x* or 3*x*), it is possible to deduce the complementary 1*x* gamete. In most Mchare-derived polyploid accessions resulting from such gametes, the parental origins of the second parent were not identified (Table 1). To learn more about the second parent, alleles from the complete genome donor (Mchare or Gros Michel) were removed from the child genotypes to deduce the gamete from the second parent. These gametes were predicted to be 1*x* except for Nadan, where the deduced gamete had two copies of chromosome 8, suggesting a 1*x* + 1 gamete. These deduced gametes were then processed as described in Martin *et al.* (2023) to obtain their genome ancestry mosaic (Supplementary Data Fig. S6). This revealed a *M. balbisiana* introgressed status of the second parents of AAB cultivars Nadan, Nendra Padaththi and Pome. The comparison of the genome ancestry mosaic of deduced gametes of these three AAB cultivars with the ancestral mosaics of A/B hybrid cultivars obtained by Martin *et al.* (2023) suggested potential similarity between their 1*x* gamete donors and two ABB triploid cultivars (Monthan and Ney Mannan; Figs 3 and 4, Supplementary Data Fig. S6). Nadan showed potential additional similarity with Saba and Pome showed potential additional similarity with Saba, Pelipita and Pisang Kelat (Supplementary Data Fig. S6). This led us to investigate the potential shared pedigree between these cultivars. As illustrated for chromosome 8 (Fig. 3A–C), for all three AAB cultivars (Pome, Nendra Padaththi and Nadan), large regions of agreement but also large regions of discordance were observed when parentage of their deduced gamete with Ney Mannan and Monthan was tested. Such patterns were visible on several chromosomes (Supplementary Data Fig. S6), suggesting close pedigree relationships. In contrast, comparison with Saba, Pelipita and Pisang Kelat did not show such large regions of agreement (Fig. 3B, C, Supplementary Data Fig. S6), excluding very close pedigree relationships.

Figure 3 also illustrates some similar agreement/discordance patterns when comparing the deduced gametes from the AAB cultivars with Ney Mannan and Monthan. This suggests that all tested AAB cultivars and the ABB Ney Mannan and Monthan are related to each other.

The mosaics of the other deduced gametes also revealed a *M. acuminata* ssp. *malaccensis* and ssp. *banksii* hybrid as second parent of Calypso and a more complex hybrid status of the second parents of Cavendish and Gros Michel (Fig. 4). As expected from validated trios, the remaining haplotype of Gros Michel matched a potential 1*x* gamete from Khai Nai On (Supplementary Data Fig. S6K). The remaining haplotype of Cavendish showed large accordance/discordance segments with Pisang Pipit or Pisang Madu as 1*x* gamete donors, suggesting that the 1*x* gamete donor of Cavendish is a close relative of both accessions (Supplementary Data Fig. S6L, M).

## DISCUSSION

We analysed the direct parentage between 178 banana individuals including 111 cultivars and 55 wild accessions and characterized parentage relationships for 7 diploid cultivars, 11 triploid cultivars and 1 tetraploid, some of them representing major subgroups of dessert banana. These results highlighted that diploid but also triploid cultivars could be parents of other diploid, triploid or tetraploid cultivars by contributing 1*x*, 2*x* or 3*x* gametes. A targeted analysis of the Mchare subgroup parentage relationships showed an important contribution of this subgroup to banana cultivars (Fig. 4).

### Mchare contributions to banana cultivars

The Mchare bananas form a phenotypically diverse subgroup of AA diploids. They are nowadays only found in some East African regions and islands, where they are particularly appreciated and culturally important (Perrier *et al.*, 2019). We found that Mchare contributed 1*x* gametes to two diploid and three triploid cultivars, and also 2*x* gametes to five triploid cultivars of our sample. It is also present in tetraploid Calypso through the Gros Michel contribution (Fig. 4). The Gros Michel dessert banana was very popular in the first half of the previous century, but being susceptible to the Panama disease (*Fusarium oxysporum* fsp. *cubense* Race 1), it was replaced by Cavendish. We confirmed that both cultivars resulted from the transmission of a 2*x* gamete from Mchare (Raboin *et al.*, 2005; Perrier *et al.*, 2009; Hippolyte *et al.*, 2012; Martin *et al.*, 2020*b*) and demonstrated that in both cases the complete Mchare genome was transmitted. In addition, we validated the implication of Mchare as 2*x* gamete donor in the AAB cultivars Nadan, Pome/Prata and Nendra Padaththi (Perrier *et al.*, 2009; Hippolyte *et al.*, 2012) with additional information on complete genome transmission, but we excluded the 2*x* contribution of Mchare to Hom Thong Mokho (Perrier *et al.*, 2009). Differences between our results and previous studies are likely due to the higher genotyping density and to the methodology that we used to validate parentage along chromosomes. However, we cannot completely exclude differences between studied individuals from distinct collections or resulting from mis-labelling. In addition, we also showed that Mchare contributed a 1*x* gamete to the triploid cultivars Mnalouki (AAB from Comoro Islands), Koja (AAA from Comoro Islands) and Pisang Ambon (AAA) and to diploid cultivars Paka (AA from East Africa) and Colatina Ouro (AA) (Fig. 4). Mchare clones have contributed their genomes to cultivars of diverse origins, including cultivars found in Africa but also triploid dessert bananas that are predicted to originate from Southeast Asia. Thus, Mchare most probably originated in Southeast Asia and were transported by humans to Africa (Perrier *et al.*, 2019).

In all cases in which the Mchare was proposed as a 2*x* gamete donor, a full genome of the Mchare was found. This is the case for the two main dessert AAA banana cultivar subgroups Cavendish and Gros Michel, and also for AAB cultivars from the Pome/Prata, Nadan and Nendra Padaththi subgroups. One can hypothesize that a complete genome of Mchare brings highly favourable agro-morphological trait combinations that could explain the selection of individuals resulting from complete Mchare gamete restitution. An alternative hypothesis is that this genome is prone to perform a full gametic restitution, thereby increasing the probability of finding such a contribution to cultivated bananas. A combination of both these hypotheses could also be considered.

### Other pedigree relationships

Second parents in Mchare-derived cultivars were various (Fig. 4). We confirmed that the second gamete present in Gros Michel was a 1*x* gamete from Khai Nai On (Raboin *et al.*, 2005; Hippolyte *et al.*, 2012). Moreover, we showed that the Galeo accession may be a grandparent of Gros Michel through Khai Nai On. We excluded Pisang Pipit or Pisang Madu as 1*x* gamete donors to Cavendish (Perrier *et al.*, 2009; Hippolyte *et al.*, 2012), although the presence of large regions consistent with the proposed relationship suggested some shared pedigree.

Other second parents to Mchare-derived cultivars included cultivars as diverse as diploid Sucrier and triploid plantains, and also unknown A/B hybrids. For AAB Nadan, Nendra Padaththi and Pome, we showed that their unknown A/B hybrid second parents were related to ABB Ney Mannan and Monthan and that they transmitted 1*x* gametes (or in the case of Nadan an aneuploid 1*x* + 1 gamete) (Fig. 4). Note that ABB cultivars from the subgroup named Bluggoe were found to be similar at the genome mosaic level to cultivars from subgroup Monthan (Supplementary Data Table S1) (Cenci *et al.*, 2021; Martin *et al.*, 2023). In this study, we also suggested that Saba cultivars derived from Monthan. Thus, the AAB cultivars Nadan, Nendra Padaththi, Pome and ABB cultivars Monthan, Ney Mannan, Bluggoe and Saba are related. This is coherent with their shared A/B recombination breakpoints on chromosome 9 (Martin *et al.*, 2023).

In this study, all identified parental relationships only involved cultivars as parents, with one exception. This can be explained by the fact that cultivars have a fixed genotype conserved through vegetative propagation, which allows the identification of natural hybridization events that occurred centuries ago. Conversely, most wild parents involved in hybridizations some centuries ago may not exist any more, due to sexual reproduction. The only exception we found is a wild individual, PKW, which was proposed as a parent of triploid cultivar Saba. The PKW accession, as well as some accessions of *M. balbisiana*, are popular in Indonesia for various uses and are vegetatively propagated (Ahmad *et al.*, 2014). This may explain why we could still identify parentage involving PKW.

### Different types of gametes and triploid individuals are involved in the formation of many banana cultivars

Several cases of transmission of un-recombined or recombined 2*x* gametes from diploid to triploid cultivars were identified. They involved mainly Mchare cultivars but also Tjau Lagada and likely Galeo. More surprisingly, in a few cases, a triploid was proposed as parent of another triploid or diploid cultivar, either as a 2*x* or as a 1*x* gamete donor. Banana cultivars, either diploid or triploid, are poorly fertile and sometimes totally sterile. Crosses have been undertaken since the 1930s in order to exploit a cultivar’s residual fertility in breeding programmes. This often required a huge number of pollinations. Cytogenetic analysis of the progenies obtained showed that diploid bananas generally produce 1*x* gametes while triploids may produce from 1*x* up to 6*x* gametes (Wilson, 1946*a*–*c*; Larter, 1948; Shepherd, 1999). These features are exploited in breeding programmes to generate tetraploids by crossing triploid cultivars with diploids. Triploids could thus also potentially be involved through natural hybridization in the formation of other cultivars. However, this should be rare given their poor levels of fertility. Based on nuclear and cytoplasmic genome information, a possible implication of triploid cultivars in the formation of other cultivars has been proposed (Carreel *et al.*, 2002; De Langhe *et al.*, 2010). Here, we identified such cases. For two triploid cultivars (ABB Saba and AAB Mnalouki), we identified a triploid parent (ABB or AAB, respectively) that could have contributed a 2*x* gamete. In addition, we observed one trio and one duo in which a 1*x* and 3*x* gamete respectively could have been provided by AAA triploids. This illustrates that such a process may occur in different types of triploid banana. However, we cannot exclude that a yet not identified diploid accession could be the parent of both the child and the triploid parent proposed in our parents–child trio analysis.

Previous studies of progeny obtained by crossing triploid Gros Michel with different diploids showed that the main class of offspring obtained was tetraploid (Wilson, 1946*c*; Larter, 1948). Based on breeding behaviour and cytological observations, it was proposed that triploid gametes were the main class of viable gametes produced by Gros Michel (Wilson, 1946*c*; Larter, 1948). Our analysis along chromosomes showed that the tetraploid cultivar Calypso resulted from the transmission of an un-recombined 3*x* gamete from the triploid Gros Michel. One region of discrepancy on chromosome 10 may be explained by aneuploidy in the triploid parent [a Gros Michel clone named Highgate (Borges *et al.*, 2014)] and/or in Calypso compared with the accession Gros Michel we have used. A triploid plantain was also previously shown to have transmitted a recombined 3*x* gamete (supplementary material in Baurens *et al.*, 2019).

Unreduced gametes are generally more frequent in interspecific hybrids and in allopolyploids, and result in most cases from disturbed meiotic processes, although premeiotic and postmeiotic doubling have also been mentioned (De Storme and Geelen, 2013; Mason and Pires, 2015). If the first division step of meiosis does not occur, meiosis is similar to mitosis and produces un-recombined unreduced gametes, retaining full parental heterozygosity (De Storme and Geelen, 2013). Other disturbances during meiosis, such as cytological alterations during the second division step [e.g. parallel spindles (d’Erfurth *et al.*, 2008)] or absence of the second division, can result in unreduced gametes with recombined chromosomes (De Storme and Geelen, 2013). The inter(sub)specific origins of cultivated bananas often resulted in the presence of large chromosomal rearrangements at a heterozygous state in their genome (Martin *et al.*, 2020*a*). This may disturb meiotic processes and/or lead to the production of non-viable, unbalanced 1*x* gametes that could favour the transmission of unreduced gametes. For example, in the case of Mchare, the presence of two independent heterozygous large reciprocal translocations (Martin *et al.*, 2020*a*) will statistically generate 75 % of unbalanced gametes during meiosis, which should be non-viable. This phenomenon mathematically increases the proportion of 2*x* gametes in the pool of viable gametes.

### Prospects for banana breeding

This study illustrated that different processes are involved in the formation of banana cultivars involving various types of gamete. They include recombined or un-recombined 2*x* gametes from diploids and various types of gametes, including 3*x* un-recombined gametes from triploids. These peculiar gametes must be rare and thus are potentially selected to conserve particularly favourable hybrid genomic combinations that required several generations to be obtained.

In this context, pre-breeding strategies that aim at maintaining such favourable genomic combinations while introgressing desirable traits such as resistance could be favoured. For example, the important contribution of the Mchare subgroup to other cultivars, often through un-recombined 2*x* gametes, suggested that its genomic combination should be preserved as much as possible. High-throughput genotyping technologies may be used to select individuals with the targeted genomic combination. These strategies, like others, will be complicated by the poor fertility of bananas but conversely may be particularly useful in this context.

## SUPPLEMENTARY DATA

Supplementary Data are available online at https://academic.oup.com/aob and consist of the following. Figure S1: flowchart and scripts used to perform parentage analyses. Figure S2: validation along chromosomes of predicted parents–child trios. Figure S3: validation along chromosomes of predicted parent–child duos and transmitted gamete types. Figure S4: Calypso Illumina reads coverage along chromosomes of DH-Pahang V4. Figure S5: analysis of Galeo contribution to cultivars. Figure S6: local proportions of SNP sites in discordance with AAB/ABB tested parentage relationships. Table S1: accession information. Table S2: list of accessions used for global trio analysis and of accessions used for identification of *M. balbisiana* and *Australimusa* private allele sites for the Mchare analysis. Table S3: global parentage trio analysis: proportions of SNP sites in accordance with trios. Table S4: global parentage trio results for synthetic *F*_1_ trios. Table S5: global analysis of Mchare contribution.

mcad065_suppl_Supplementary_Materials

mcad065_suppl_Supplementary_Tables
