# Supplementary material for: Shared pedigree relationships and transmission of unreduced gametes in cultivated banana
Source: Ann Bot. 2023 Jun 2;131(7):1149–61. doi: 10.1093/aob/mcad065 (PMC10457027; doi:10.1093/aob/mcad065)

**Shared pedigree relationships and transmission of unreduced gametes in cultivated banana**

Guillaume Martin, Franc-Christophe Baurens, Karine Labadie, Catherine Hervouet, Frédéric Salmon, Franck Marius, Nilda Paulo-de-la-Reberdiere, Ines Van den Houwe, Jean-Marc Aury, Angélique D’Hont, Nabila Yahiaoui.

**SUPPLEMENTARY INFORMATION**

TABLE OF CONTENT:

SUPPLEMENTARY FIGURES:

Fig. S1: Flowchart and scripts used to perform parentage analyses.

Fig. S2: Validation along chromosomes of predicted parent-child trios.

Fig. S3: Validation along chromosomes of predicted parent child duos and transmitted gamete types.

Fig. S4: Calypso Illumina reads coverage along chromosomes of DH-Pahang V4.

Fig. S5: Analysis of Galeo contribution to cultivars.

Fig. S6: Local proportions of SNP sites in discordance with AAB/ABB tested parentage relationships.

SUPPLEMENTARY TABLES (see associated Excel file):

Table S1: Accession information.

Table S2: List of accessions used for global trio analysis and of accessions used for identification of *M. balbisiana* and Australimusa private alleles sites for the Mchare analysis.

Table S3: Global parentage trio analysis: proportions of SNP sites in accordance with trios.

Table S4: Global parentage trio results for synthetic F1 trios.

Table S5: Global analysis of Mchare contribution.

SUPPLEMENTARY FIGURES


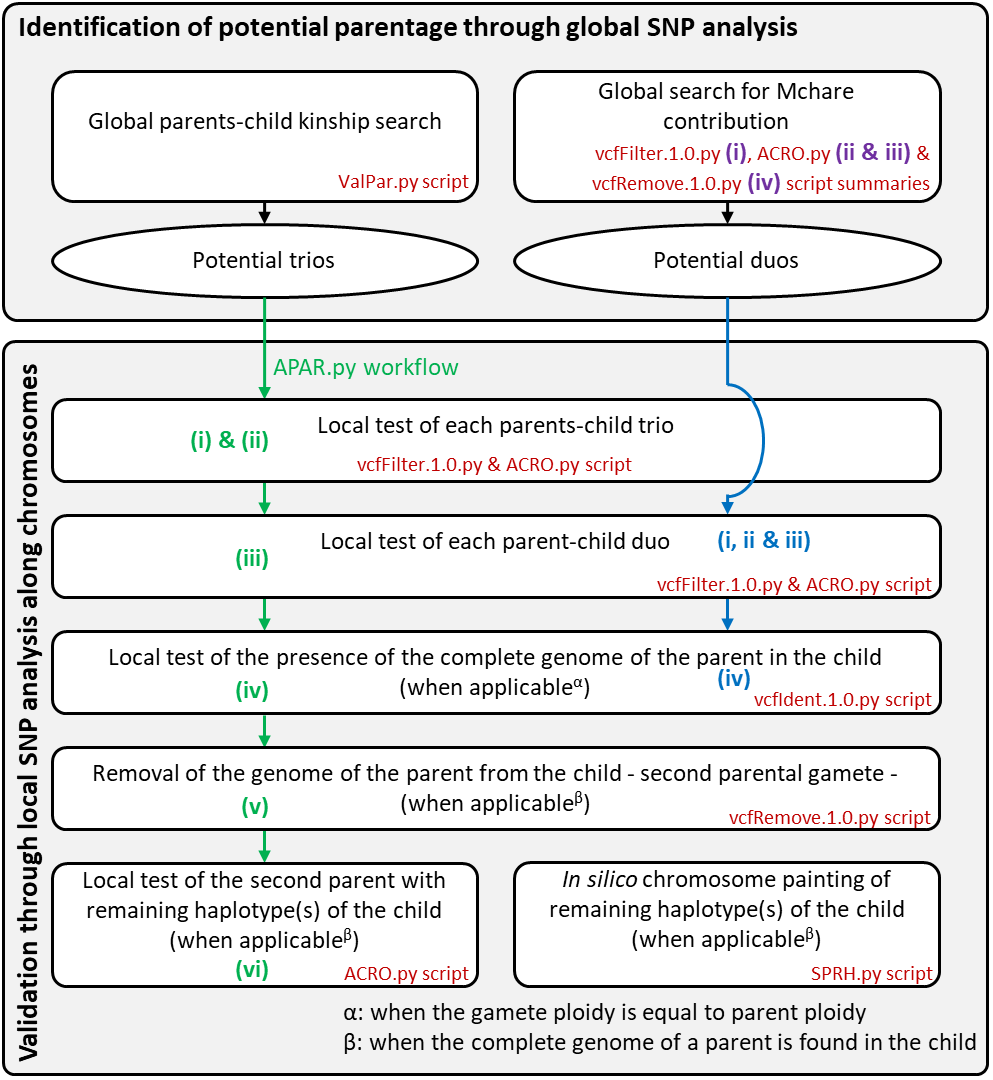
**Fig. S1: Flowchart and scripts used to perform parentage analyses.** Analyses are grouped in two categories: the identification of potential parentage through global SNP analysis and the validation of potential parentage through local SNP analysis along chromosomes. Scripts (in red) used in workflows (purple, green and blue) are indicated. Numbers (i, ii, iii, iv, v and vi) refer to steps described in material and methods corresponding sections.


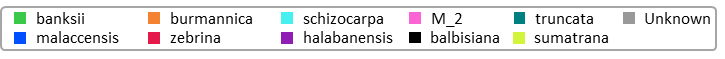

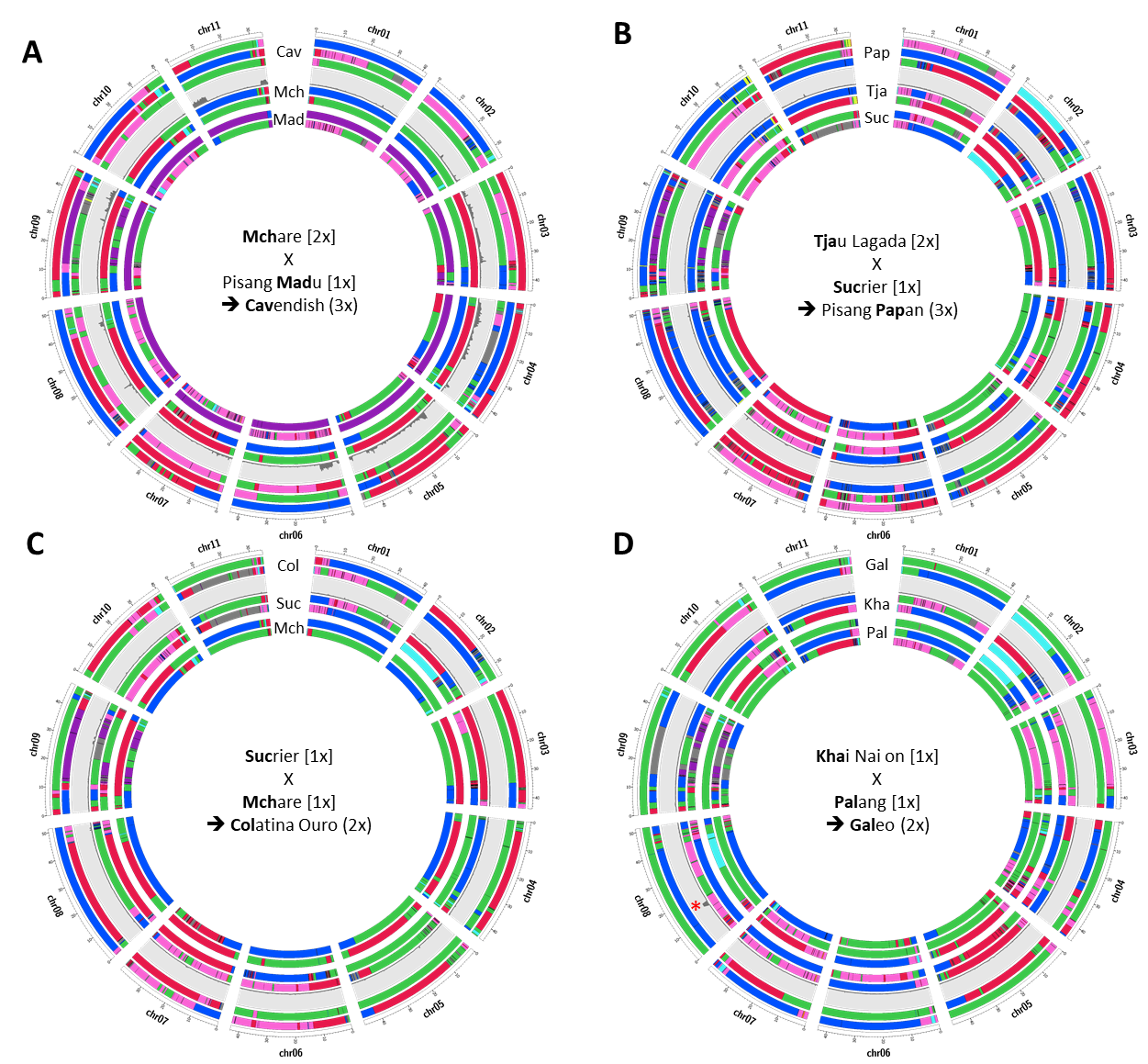
**Fig. S2: Validation along chromosomes of predicted parent-child trios.** Accession chromosome ancestry mosaics obtained from Martin et al. (2023) and the local proportion of sites in discordance with tested trios are represented**.** Child pseudo-haplotypes are represented on outer circles and are separated from tested parents’ pseudo-haplotypes (on inner circles) by the local proportion of alleles in discordance with the tested parentage (value between 0 and 1). Ploidy of the parental tested gamete is indicated between square brackets and the ploidy of the child is indicated between brackets. In the legend, colour codes “banksii”, “burmannica”, “zebrina”, “malaccensis”, “truncata”, “sumatrana” and “halabanensis” stand for an origin from *M. a.* ssp *banksii/microcarpa/errans*, *burmannica*, *zebrina*, *malaccensis*, *truncata*, *sumatrana* and *halabanensis* respectively. “schizocarpa” and “balbisiana” stand for *Musa schizocarpa* and *M.* *balbisiana* respectively. “M_2” is an uncharacterized contributor to banana and “Unknown” corresponds to regions in which no origin could be attributed. **A**, **B**, **C**, **D**, **E**, **F**, **G**, **H** and **I** correspond to nine tested trios whose names are indicated in each figure centre.


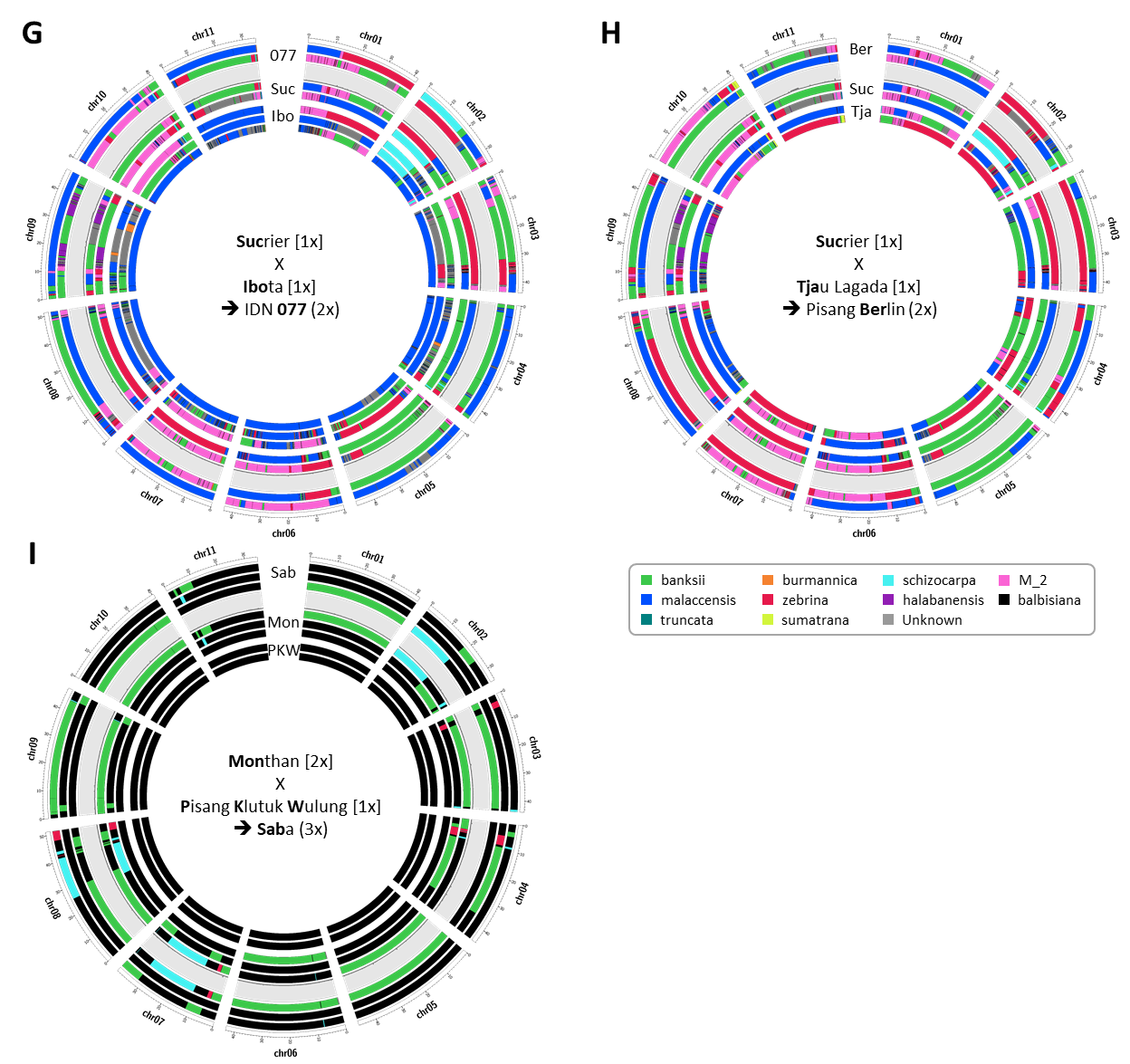

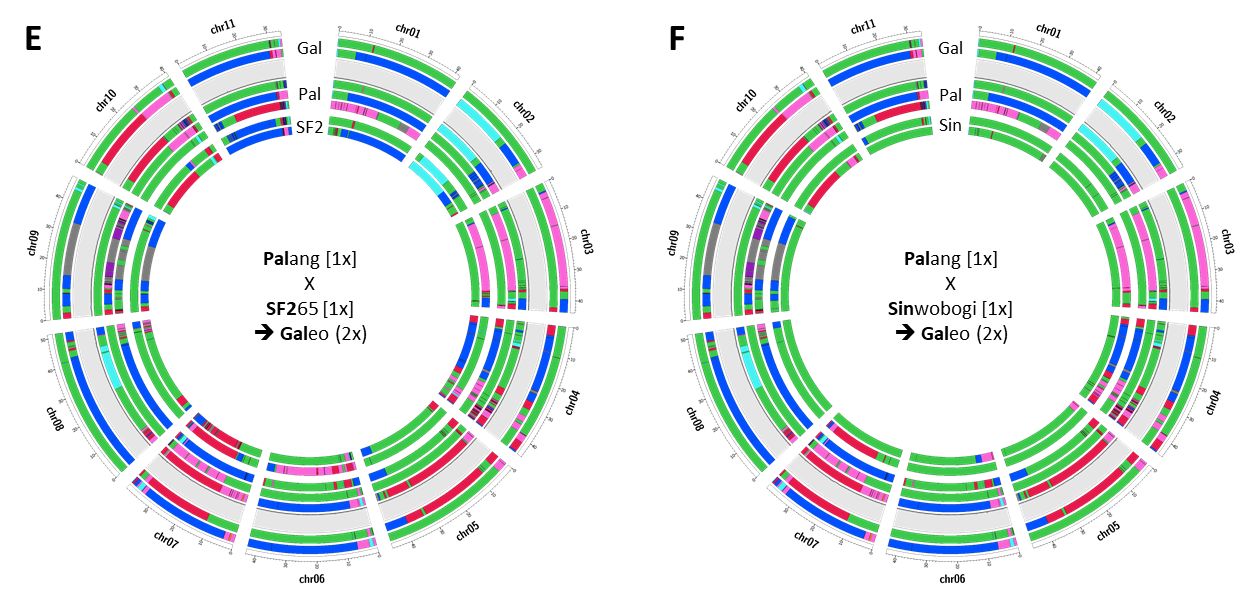


**Fig. S3: Validation along chromosomes of predicted parent child duos and transmitted gamete types.** Accession chromosome ancestry mosaics obtained from Martin et al. (2023) and the local proportion of sites in discordance with tested duos and gamete types are represented**.** Child pseudo-haplotypes are represented on outer circles and are separated from tested parent pseudo-haplotypes (on inner circles) by the local proportion of alleles in discordance with tested parentage (value between 0 and 1). Depending on the duos tested, different type of parentage could be tested: a haploid gamete restitution (1x), a diploid gamete restitution (2x) or a diploid gamete with complete genome restitution (2x^c^). Ploidy of the parental tested gamete is indicated between square brackets and the ploidy of the child is indicated between brackets. In the legend, colour codes “banksii”, “burmannica”, “zebrina”, “malaccensis”, “truncata”, “sumatrana” and “halabanensis” stand for an origin from *M. a.* ssp *banksii/microcarpa/errans*, *burmannica*, *zebrina*, *malaccensis*, *truncata*, *sumatrana* and *halabanensis* respectively. “schizocarpa” and “balbisiana” stand for *Musa schizocarpa* and *M.* *balbisiana* respectively. “M_2” is an uncharacterized contributor to banana and “Unknown” corresponds to regions in which no origin could be attributed. **A**, **B**, **C**, **D**, **E**, **F**, **G**, **H**, **I**, **J** and **K** correspond to eleven tested duos whose names are indicated in each figure centre.


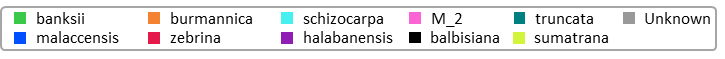
**
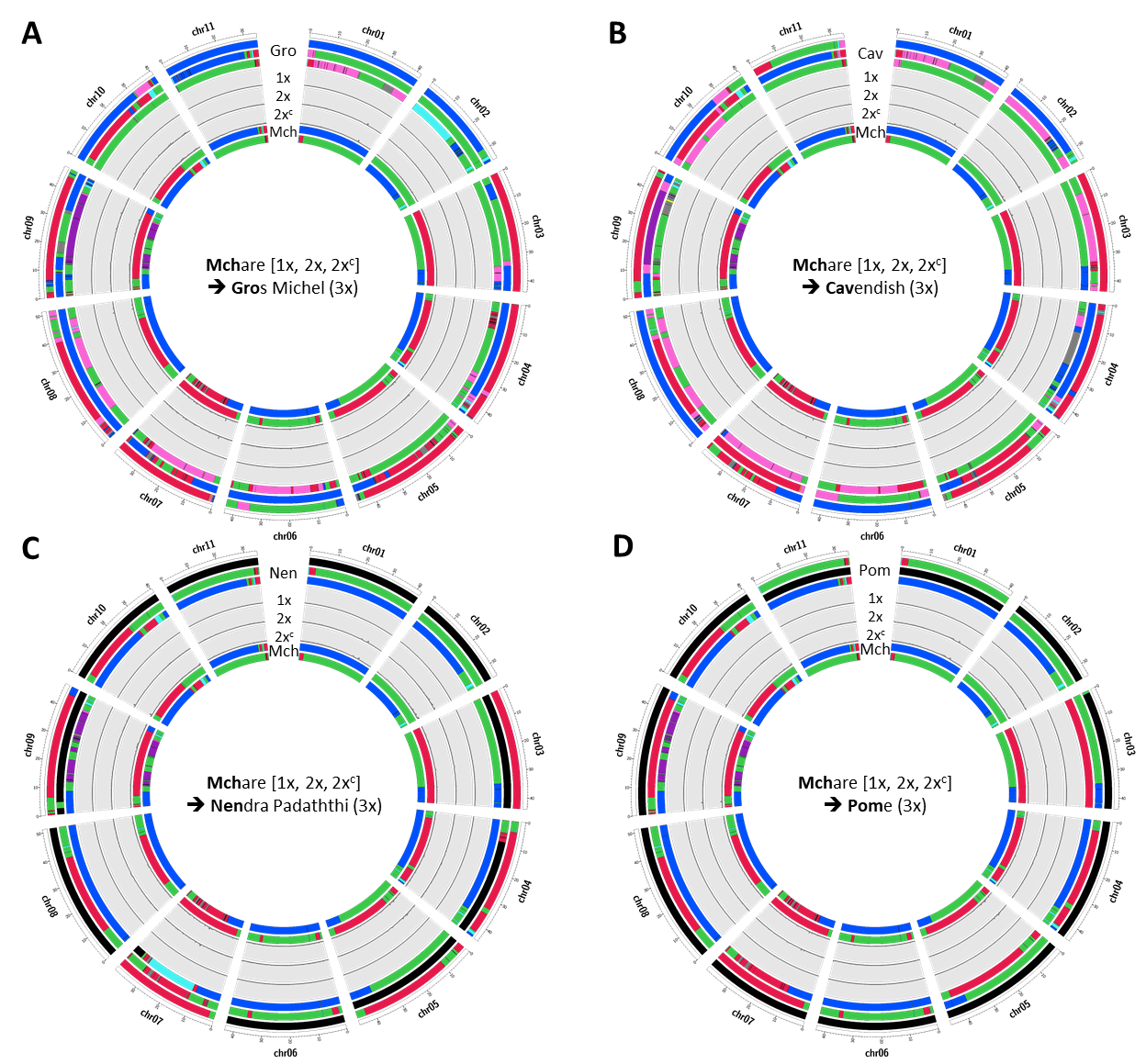
**


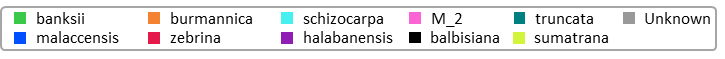

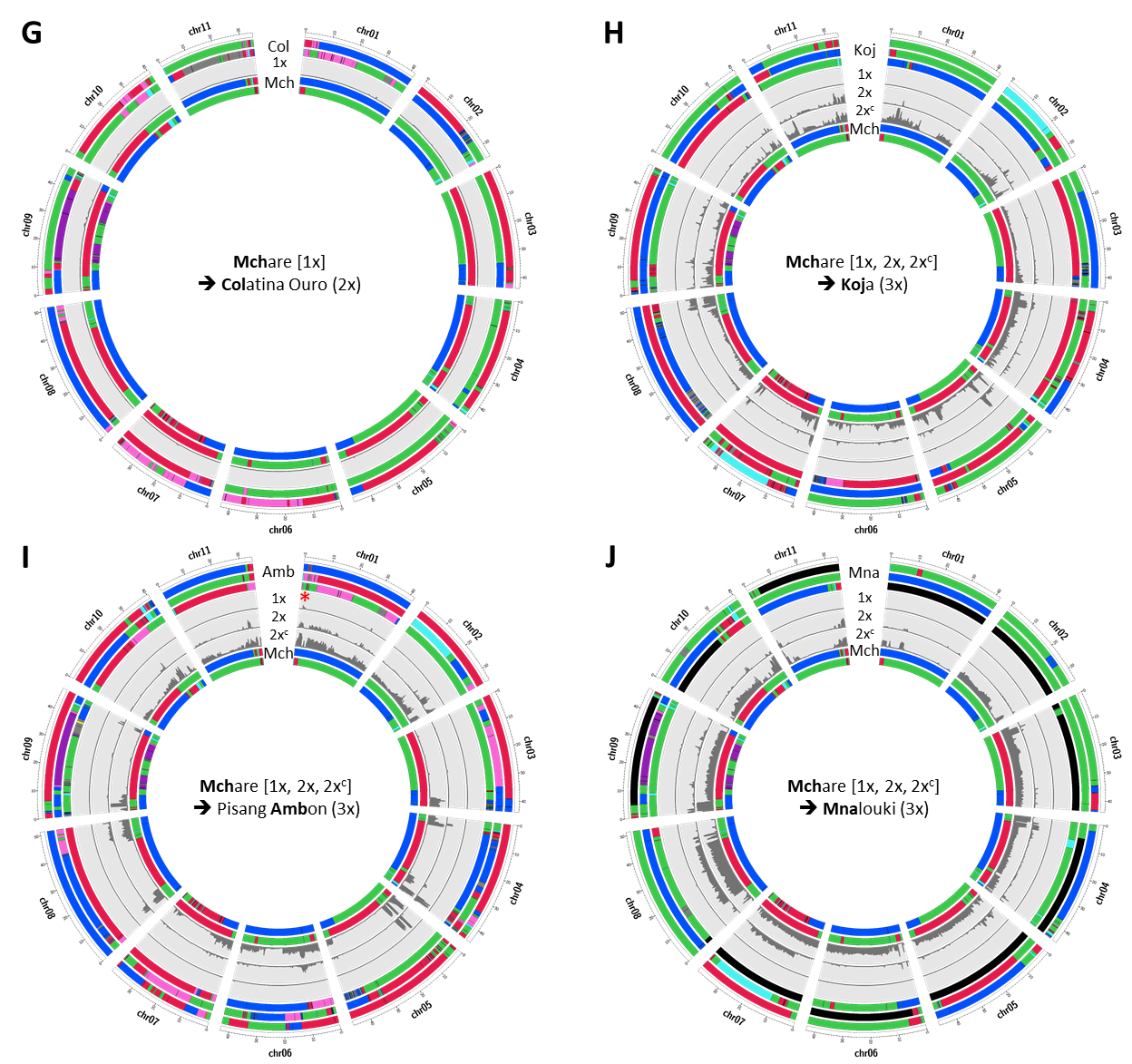

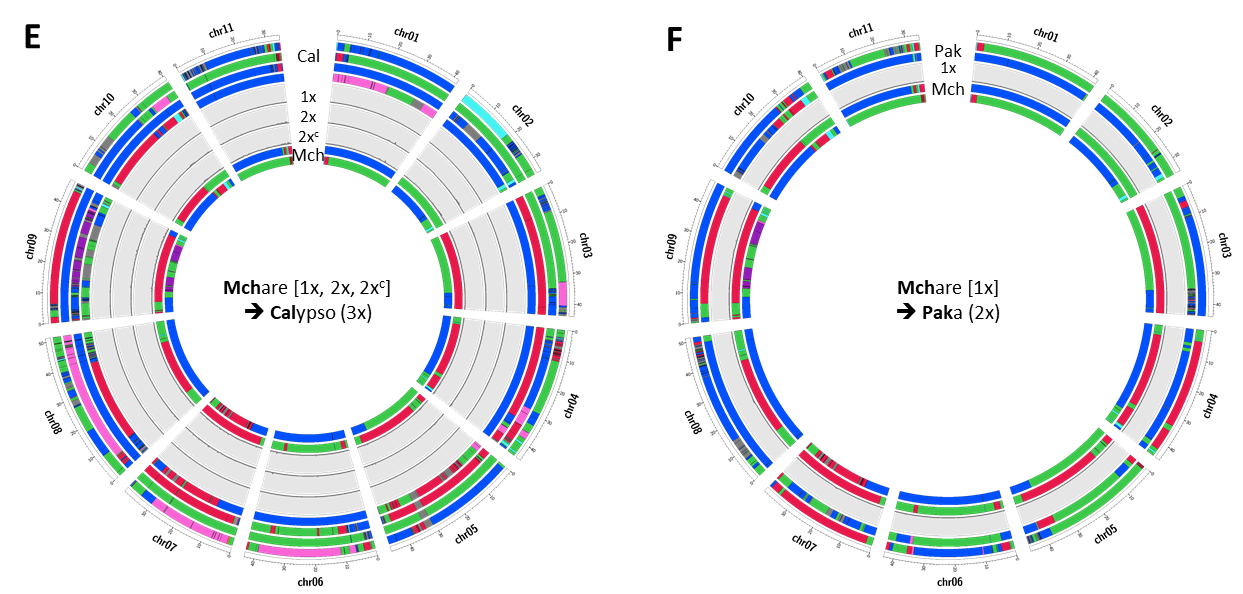


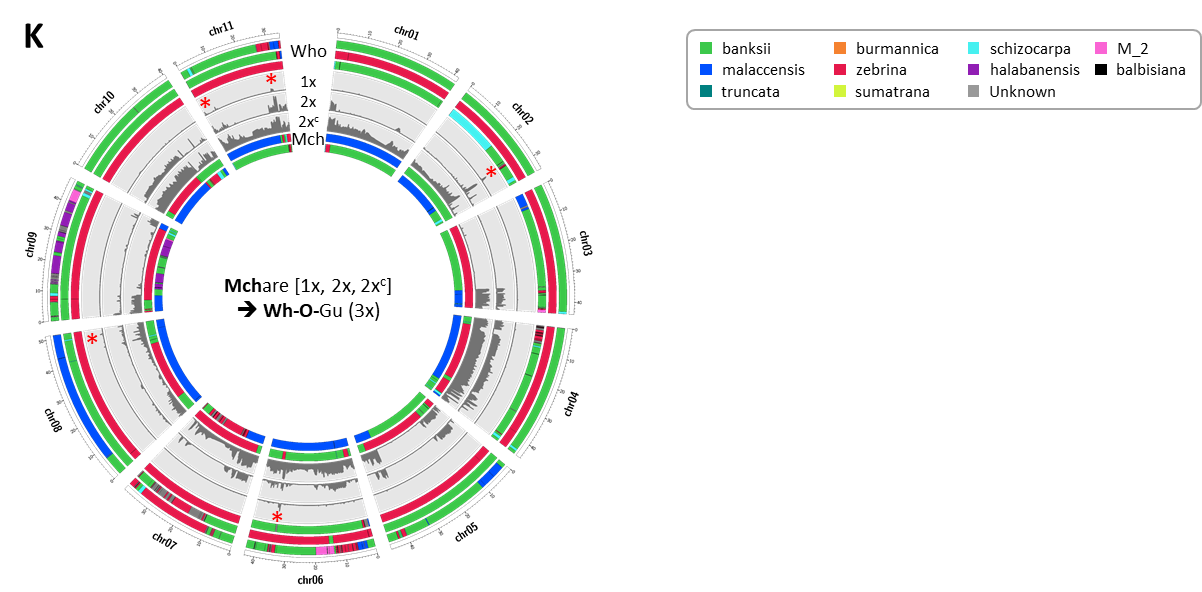


**Fig. S4: Calypso Illumina reads coverage along chromosomes of DH-Pahang V4.** The visualisation is performed using the vcf2cov.py program added to vcfhunter toolbox (<https://github.com/SouthGreenPlatform/VcfHunter>). Each blue dot represents the Calypso Illumina read coverage of each site reported in the vcf. The red curve represents the mean coverage calculated on sliding windows of 401 SNPs. The black lines at the centre of each graph represent the median coverage observed for Calypso. Black boxes located two regions of aneuploidy identified in Calypso according to deviation from expected read coverage.


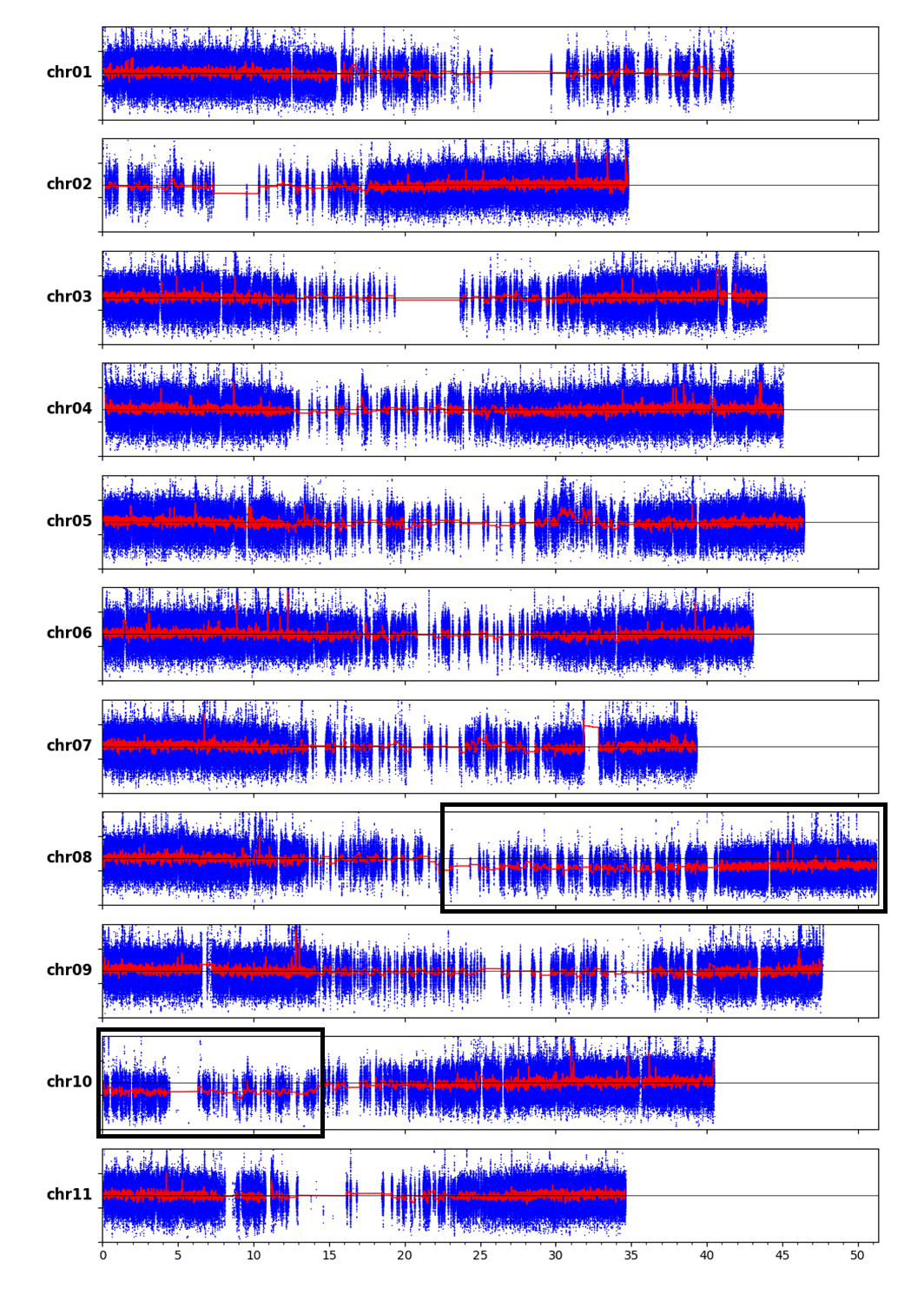


**Fig. S5: Analysis of Galeo contribution to cultivars. A)** Circular representation validating the presence of a complete genome of Galeo into the triploid Palang accession. Palang pseudo-haplotypes are represented on outer circles and are separated from tested Galeo pseudo-haplotypes (on inner circles) by the local proportion of alleles in discordance with the tested parentage (value between 0 and 1). Haploid (1x), diploid (2x) and complete genome (2x^c^) gamete restitutions were tested. Ploidy of the tested parental gamete is indicated between square brackets and the ploidy of the child is indicated between brackets. Accession chromosome ancestry mosaics were obtained from (Martin et al., 2023). In the legend, colour codes “banksii”, “burmannica”, “zebrina”, “malaccensis”, “truncata”, “sumatrana” and “halabanensis” stand for an origin from *M. a.* ssp *banksii/microcarpa/errans*, *burmannica*, *zebrina*, *malaccensis*, *truncata*, *sumatrana* and *halabanensis* respectively. “schizocarpa” and “balbisiana” stand for *Musa schizocarpa* and *M.* *balbisiana* respectively. “M_2” is an uncharacterized contributor to banana and “Unknown” corresponds to regions in which no origin could be attributed. **B)** Proposed scenario of Galeo’s contribution explaining the first identified trios. Galeo likely contributed a complete genome to triploid cultivar Palang (black and grey haplotypes) and a 1x recombined gamete to each of diploid cultivars Khai Nai On, Sinwobogi and SF265 (grey/black haplotype). Dashed boxes symbolise the 1x haplotype obtained from the other unknown parent. Because a complete genome of Galeo is found into Palang, Palang combined with either Khai Nai On, Sinwobogi or SF265 could give Galeo as child as it could provide a recombined gamete (framed in dark grey) complementing the haplotypes found in Khai Nai On, Sinwobogi or SF265 (framed in light grey).


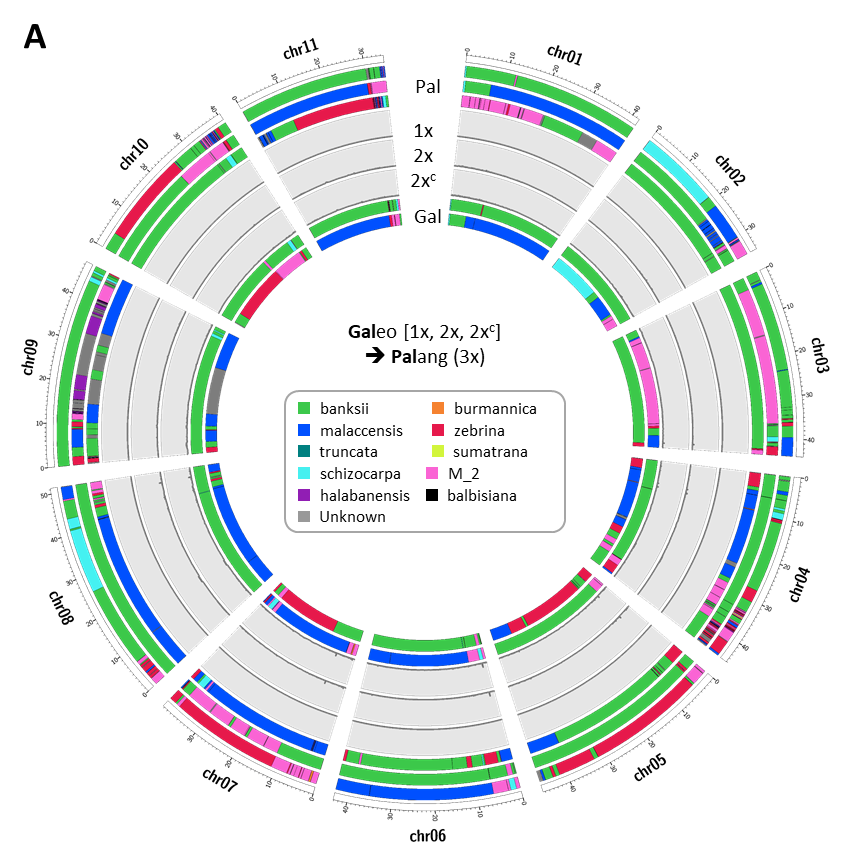


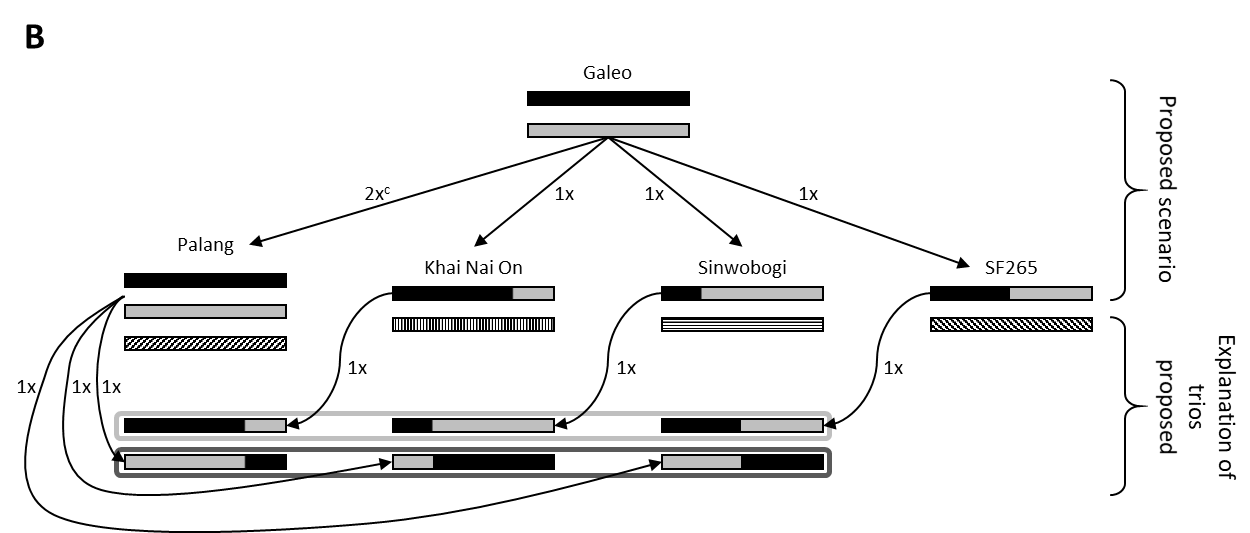


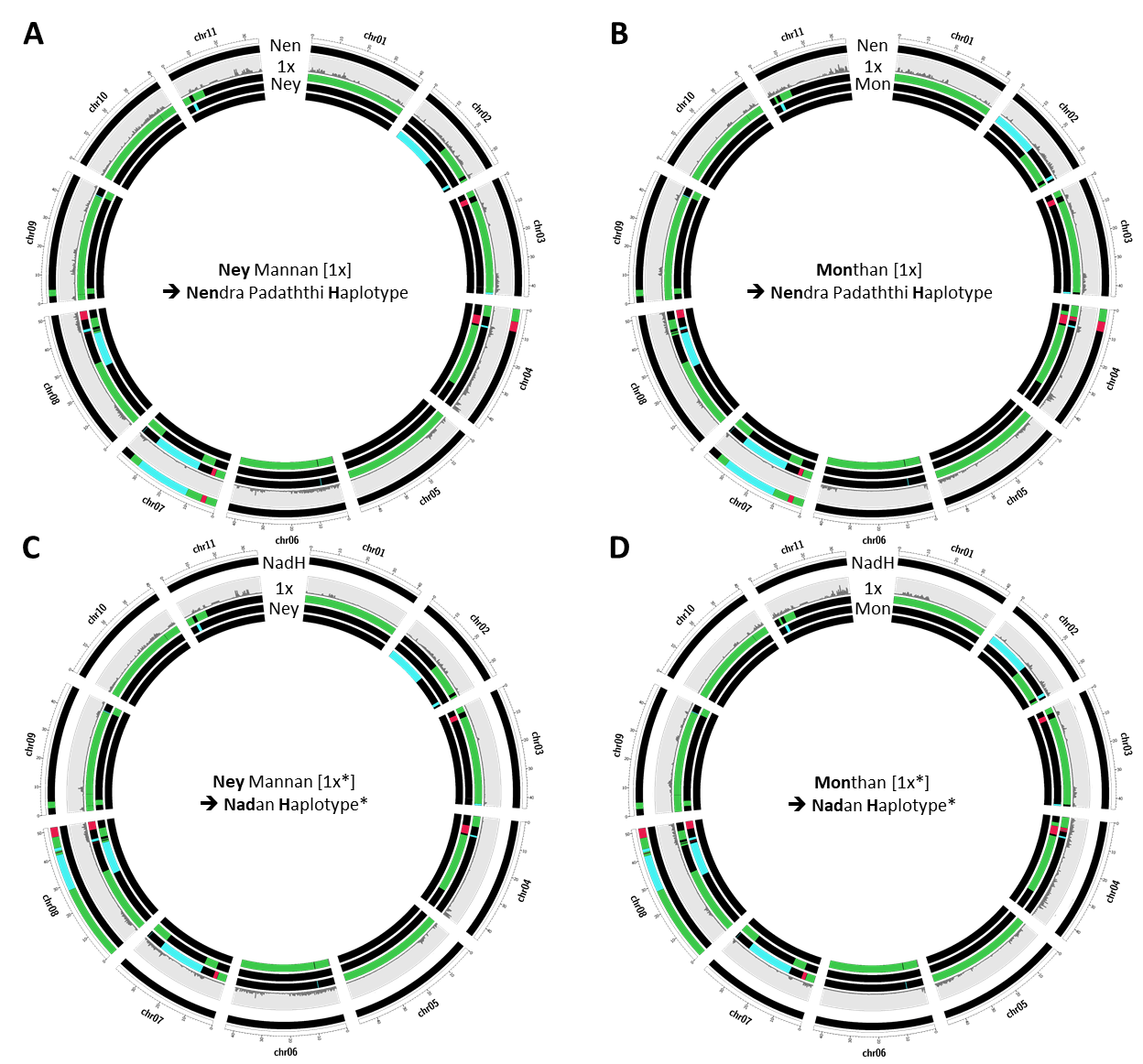

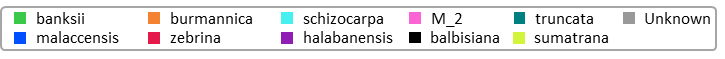
**Fig. S6: Local proportions of SNP sites in discordance with AAB/ABB tested parentage relationships.** Analysis was performed on deduced gametes from Nendra Padaththi (**A** and **B**), Nadan (**C** to **E**), Pome (**F** to **J**), Gros Michel (**K**) and Cavendish (**L** and **M**) after removal of the Mchare genotype that has been found complete in these individuals. Chromosome painting was performed according to Martin et al., 2023 on the deduced gamete. Deduced gamete(s) (pseudo-)haplotypes are represented on outer circles and are separated from tested parent pseudo-haplotypes (on inner circles) by the local proportion of alleles in discordance with tested parentage (value between 0 and 1). Ploidy of the parental tested gamete is indicated between square brackets and the ploidy of the child is indicated between brackets. In the legend, colour codes “banksii”, “burmannica”, “zebrina”, “malaccensis”, “truncata”, “sumatrana” and “halabanensis” stand for an origin from *M. a.* ssp *banksii/microcarpa/errans*, *burmannica*, *zebrina*, *malaccensis*, *truncata*, *sumatrana* and *halabanensis* respectively. “schizocarpa” and “balbisiana” stand for *Musa schizocarpa* and *M.* *balbisiana* respectively. “M_2” is an uncharacterized contributor to banana and “Unknown” corresponds to regions in which no origin could be attributed. **A**, **B**, **C**, **D**, **E**, **F**, **G**, **H**, **I**, **J**, **K**, **L** and **M** correspond to thirteen tested trios whose names are indicated in each figure centre.


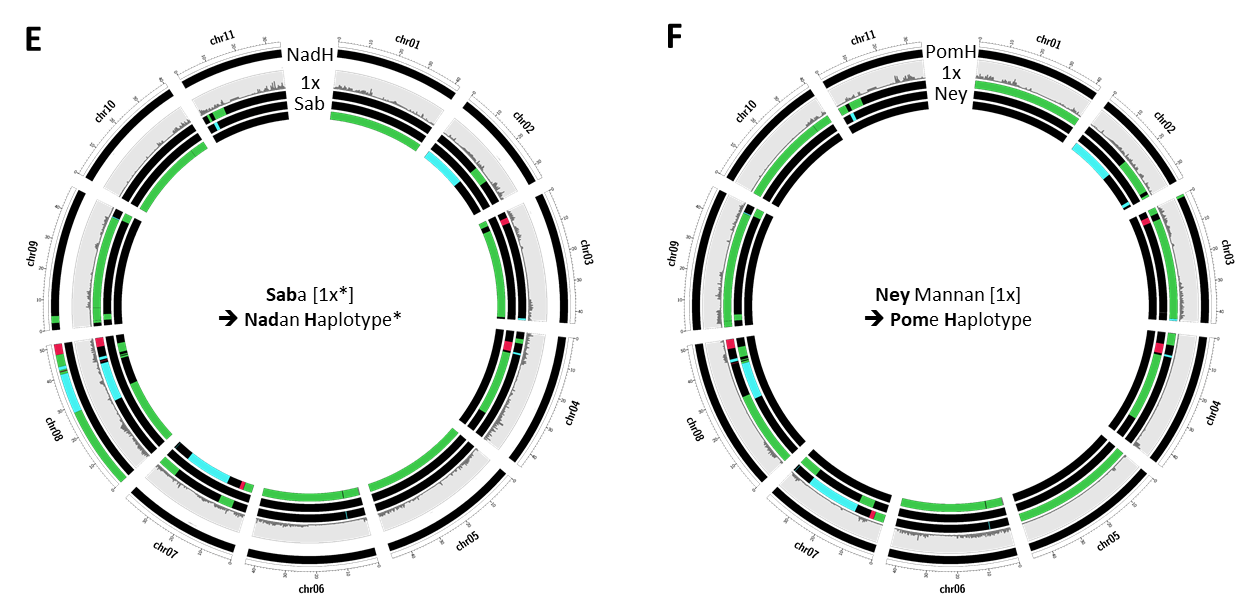

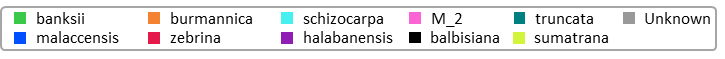

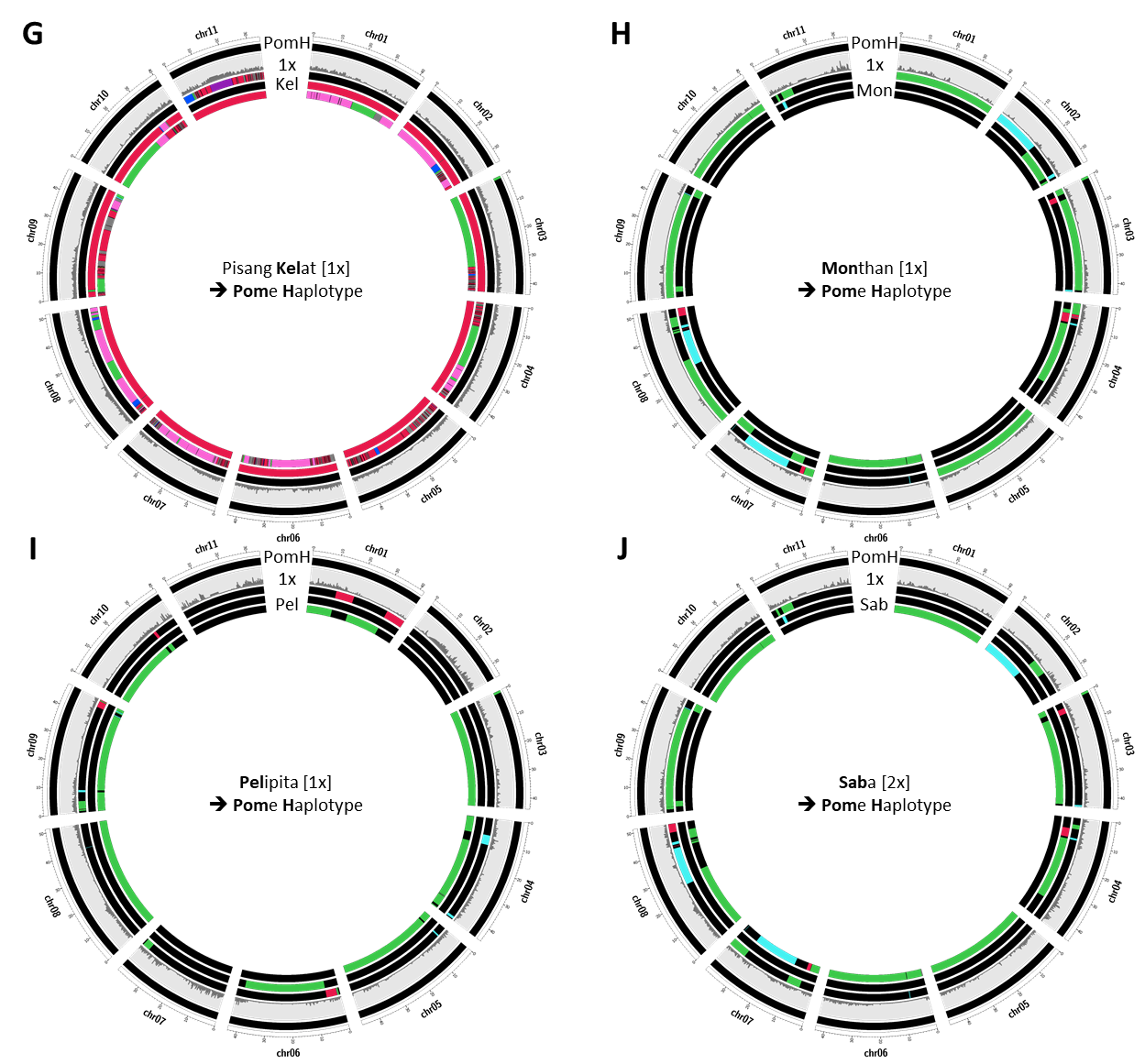


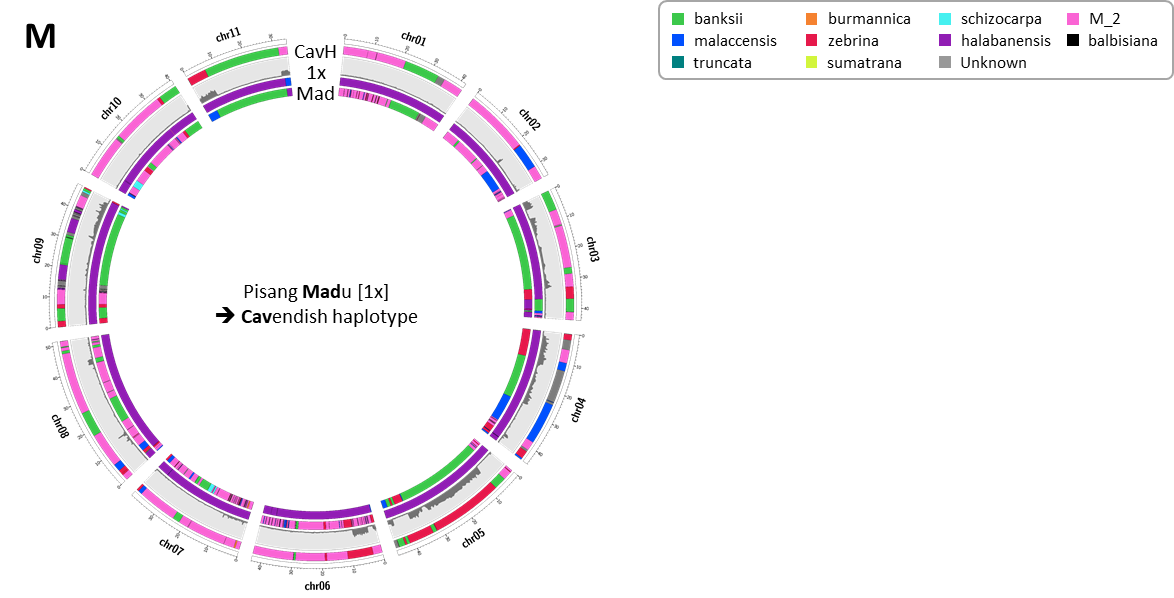

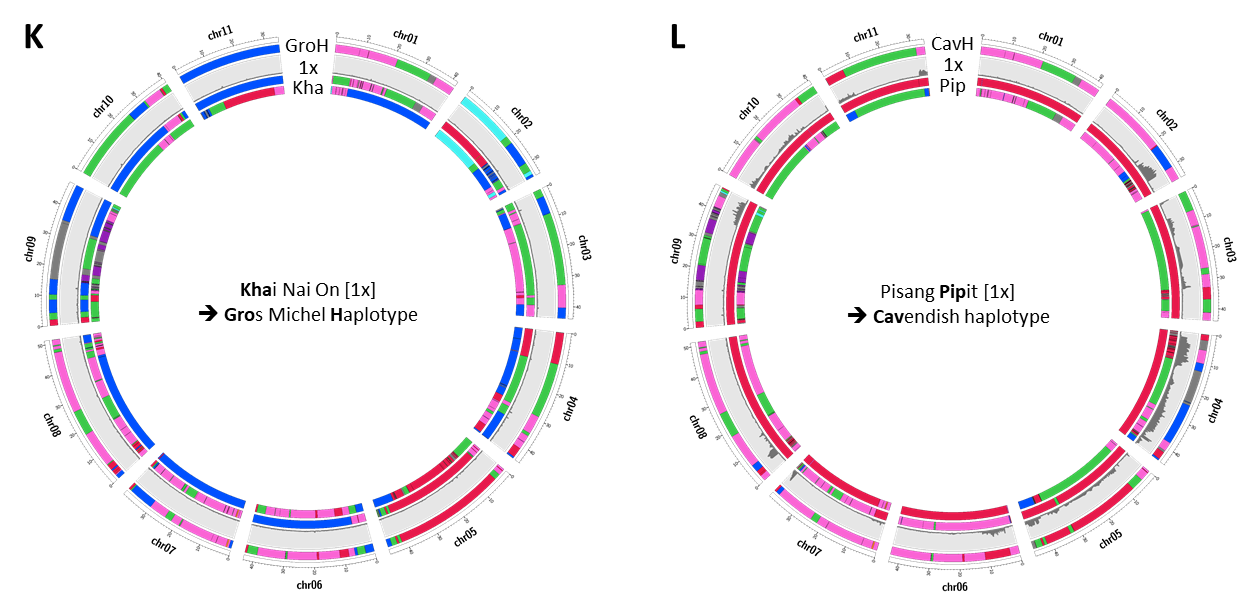

Supplement: mcad065_suppl_Supplementary_Materials [file mcad065_suppl_supplementary_materials.docx]
